# Inducible protein degradation reveals inflammation-dependent function of the Treg cell lineage-defining transcription factor Foxp3

**DOI:** 10.1126/sciimmunol.adr7057

**Published:** 2025-06-06

**Authors:** Christina Jäger, Polina Dimitrova, Qiong Sun, Jesse Tennebroek, Elisa Marchiori, Markus Jaritz, Rene Rauschmeier, Guillem Estivill, Anna Obenauf, Meinrad Busslinger, Joris van der Veeken

**Affiliations:** 1 Research Institute of Molecular Pathology (IMP), Vienna BioCenter (VBC), Campus-Vienna-Biocenter 1, A-1030 Vienna, Austria; 2 Vienna BioCenter PhD Program, Doctoral School of the University of Vienna and Medical University of Vienna, 1030 Vienna, Austria

## Abstract

Regulatory T (Treg) cells are immunosuppressive CD4 T cells defined by expression of the transcription factor Foxp3. Genetic loss-of-function mutations in *Foxp3* cause lethal multi-organ autoimmune inflammation resulting from defects in Treg cell development and suppressive activity. Whether Treg cells are continuously dependent on Foxp3 is still unclear. Here, we leveraged chemically-induced protein degradation to show that functionally suppressive Treg cells in healthy organs can persist in the near-complete absence of Foxp3 protein for at least 10 days. Conversely, Treg cells responding to type 1 inflammation in settings of autoimmunity, viral infection, or cancer were selectively lost upon Foxp3 protein depletion. Acute degradation experiments revealed that Foxp3 acts mostly as a direct transcriptional repressor and modulates responsiveness to cytokine stimulation. This inflammation-dependent requirement for continuous Foxp3 activity enabled induction of a selective anti-tumor immune response upon systemic Foxp3 depletion, without causing deleterious T cell expansion in healthy organs.

## Introduction

Regulatory T (T_REG_) cells are an essential immunosuppressive CD4 T cell lineage defined by expression of the X-linked, forkhead box transcription factor Foxp3 (1). T_REG_ cells are present in most tissues and continuously required to suppress the activation and expansion of self-reactive conventional T (T_CON_) cells (1). T_REG_ cells can also infiltrate tumors where they inhibit anti-tumor T cell responses and promote disease progression (2–7). Systemic T_REG_ cell ablation effectively inhibits tumor growth in mice but also causes severe autoimmunity. Clinical translation of T_REG_ cell-based immunotherapies therefore hinges on the development of strategies to selectively deplete T_REG_ cells from tumors but not other organs (8).

Genetic loss-of-function mutations in *Foxp3* cause lethal multi-organ autoimmunity resulting from defects in T_REG_ cell-suppressive activity, fitness, and lineage stability (9–15). However, whether T_REG_ cells are continuously dependent on Foxp3 is less clear. Deletion of a *lox*P-flanked *Foxp3* allele (*Foxp3*^*flox*^) using tamoxifen-inducible Cre recombinase caused an approximately 2-fold expansion of splenic CD4 T cells after 4 weeks, a much more modest phenotype than that of mice constitutively lacking *Foxp3* (16). However, because the *Foxp3*^*flox*^ allele lacks a fluorescent reporter that would enable direct identification of *Foxp3*-transcribing cells after the deletion of essential exons, it is unclear if lineage-committed T_REG_ cells are maintained in this setting. Another study used retrovirally-expressed Cre recombinase to induce *Foxp3* deletion before transferring T_REG_ cells together with wildtype CD4 T cells into lymphopenic mice (11). In this setting, T_REG_ cells downregulated non-deleted portions of the *Foxp3* transcript as well as other T_REG_ cell signature genes, suggesting that the transferred T_REG_ cells quickly de-differentiated upon loss of Foxp3. Whether this strict and continuous requirement for Foxp3 also applies to T_REG_ cells residing in their native tissue environments or within tumors is still unclear.

Foxp3 binds to approximately 10,000 regions in the genome and interacts with hundreds of other nuclear proteins (17–20). How these events impact gene expression has been difficult to define. Genetic deficiency in *Foxp3* affects the transcription of a subset of Foxp3-bound genes; however, many genes that are not bound by Foxp3 also change their expression. Foxp3 can thus be assumed to drive both direct and indirect transcriptional changes. Indirect changes may arise as a secondary consequence of direct changes in the expression of genes encoding signaling molecules, transcription factors, co-factors, and other factors that impact the T_REG_ cell transcriptome (19). Importantly, indirect regulation may also affect genes that are bound by Foxp3, which can therefore not be assumed to represent direct transcriptional targets. It is therefore still unclear if Foxp3 acts as a direct transcriptional activator, a repressor or both, and exactly which genes it regulates (17–23). Similarly, while Foxp3-containing protein complexes can encompass both transcriptional co-activators and co-repressors, it is unknown which of these are important for direct target gene regulation (20, 21, 24, 25).

T_REG_ cell functionality is extensively modulated by environmental stimuli, including antigen, co-stimulatory molecules, cytokines, chemokines, alarmins, hormones, and xenobiotics (26, 27). Transcription factors and co-factors whose activity or expression is induced by these stimuli can alter the T_REG_ cell transcriptome and epigenome and direct cell migration, proliferation, and suppressive functions (28). Some of these factors can also physically interact with Foxp3 or bind to the same sites in the genome (17, 19–21, 24). This raises the possibility that the direct target genes regulated by Foxp3 are context-dependent, and that a differential requirement for Foxp3 may exist in T_REG_ cells exposed to inflammatory environments, such as a tumor, compared to T_REG_ cells residing in healthy organs.

Here, we developed a genetically modified mouse model that enables rapid chemically-induced degradation of Foxp3 protein in fluorescently-labeled T_REG_ cells. Using this system, we found that *Foxp3*-expressing cells in lymphoid and non-lymphoid tissues of healthy mice were largely maintained and continued to suppress T cell expansion following the near-complete depletion of Foxp3 protein for 10 days. In contrast, a specialized subset of T-bet^+^CXCR3^+^ T_REG_ cells that arises in settings of autoimmunity, viral infection, or cancer was continuously dependent on Foxp3 for its maintenance. Acute Foxp3 degradation revealed its function as an activation-dependent transcriptional repressor, capable of modulating responses to cytokine stimulation. This inflammation-dependent requirement for continuous Foxp3 activity enabled induction of a selective anti-tumor immune response upon systemic Foxp3 protein depletion, without causing deleterious T cell expansion in healthy organs.

## Results

### Chemically-induced Foxp3 protein degradation in mice

**Fig. 1:**
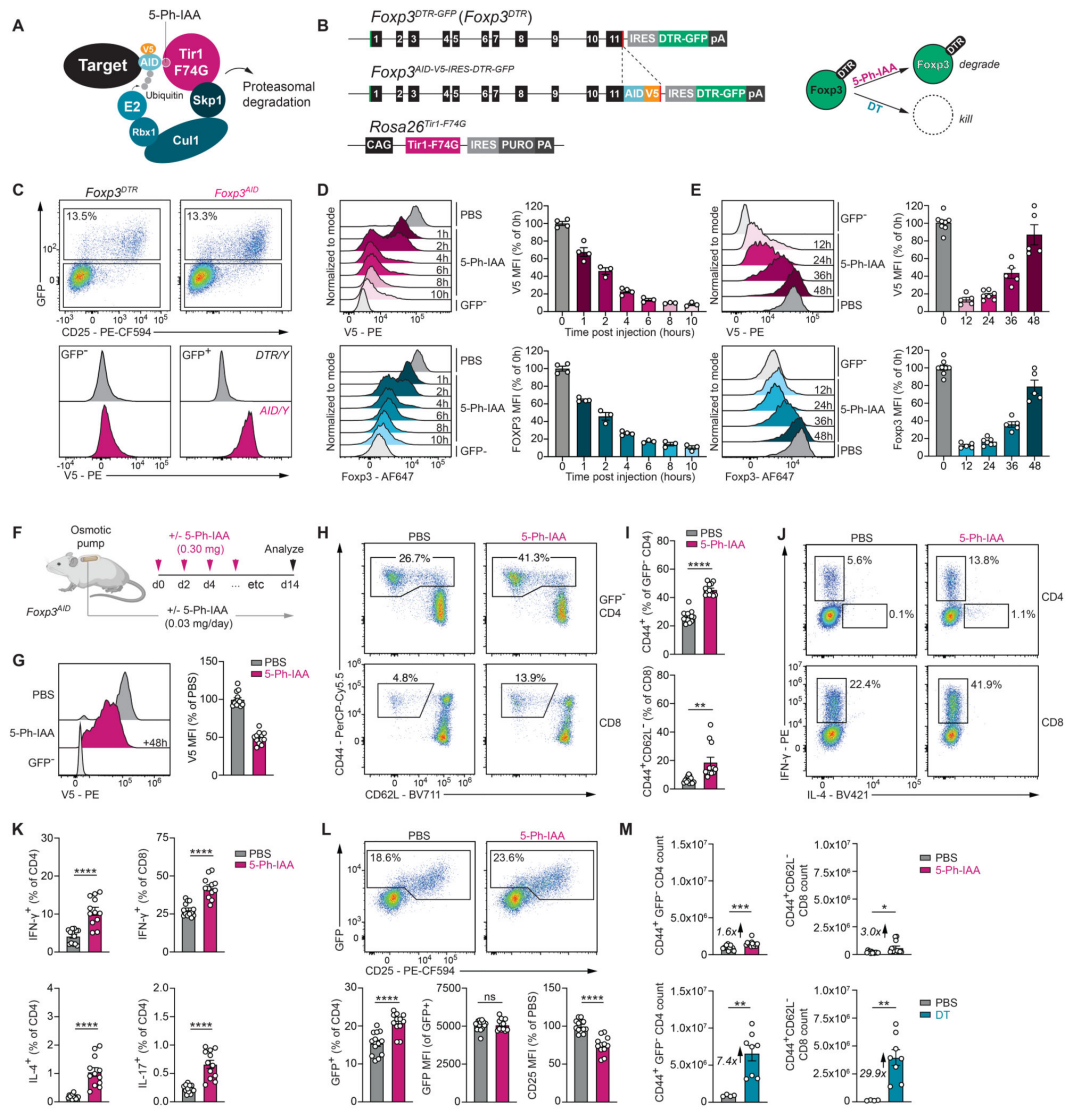
Chemically-induced degradation of Foxp3. (**A**) Schematic illustration of the AID2 system. (**B**) Schematic illustration of the *Foxp3*^*AID-V5-IRES-DTR-GFP*^ and *Rosa26*^*Tir1-F74G*^ alleles and their function. (**C**) Representative flow cytometry plots showing GFP fluorescence and V5 staining in the spleen. (**D**) Flow cytometry measurements of Foxp3 degradation kinetics following i.p. injection of 5-Ph-IAA. Geometric mean fluorescence intensity (MFI) of V5 or Foxp3 intracellular antibody staining in GFP^+^ T_REG_ cells was normalized to the average of PBS-treated control samples after subtracting background fluorescence from the GFP^-^ CD4 T cell population. Pooled data from two independent experiments a total of 3 to 4 mice per timepoint. (**E**) Foxp3 protein recovery after 5-Ph-IAA injection. Pooled data from three independent experiments with 5-8 mice per timepoint (**F**) Experimental setup. Osmotic pumps delivering ∼0.03 mg/day 5-Ph-IAA or PBS were implanted into male and female *Foxp3*^*AID*^ mice. 0.3 mg 5-Ph-IAA or PBS was injected every 48 hours for 14 days. (**G**) Foxp3-V5 levels 48 hours after the final 5-Ph-IAA injection. (**H-I**) Flow cytometry analysis of CD44 and CD62L on splenic CD4 and CD8 T cells after 14 days of continuous 5-Ph-IAA treatment. (**J-K**) Cytokine production measured by intracellular staining and flow cytometry. (**L**) GFP and CD25 levels on splenic CD4 T cells following 14-day continuous Foxp3 protein depletion. (**M**) Total activated GFP^-^ CD4 and CD8 T cell counts after 14 days of Foxp3 protein degradation (top) or 11 days after two consecutive daily injections of DT (bottom). Panels G-M show pooled data from three independent experiments with 12-13 mice per group (Foxp3 degradation) or pooled data from two independent experiments with 4-8 mice per group (T_REG_ cell depletion). Error bars show mean with SEM. *P* values were calculated using unpaired two-tailed Student’s *t*-test. ns: *P* > 0.05, *: *P* ≤ 0.05, **: *P* ≤ 0.01, ***: *P* ≤ 0.001, ****: *P* ≤ 0.0001.

To study the continuous requirement for Foxp3 in mature T_REG_ cells, we employed the auxin-inducible degron (AID) system, which leverages the *Oryza sativa* Tir1 adaptor protein (29). Tir1 mediates chemically-induced protein degradation by recruiting a Skp1-Cul1-F-box E3 ligase complex to an AID peptide-tagged target protein in response to the plant hormone auxin (indole-3-acetic-acid, IAA) (Fig. 1A). The application of the AID system *in vivo* has been limited by high levels of background degradation (30). A recently developed “AID2” system overcomes this limitation by introducing a phenylalanine-to-glycine point mutation in Tir1-F74G, which diminishes binding to IAA and instead enables binding to a synthetic high-affinity ligand, 5-phenol-indole-3-acetic-acid (5-Ph-IAA) (29), which we employ here.

To enable auxin-inducible Foxp3 protein degradation, we generated a *Foxp3*^*AID-V5-IRES-DTR-GFP*^ allele, expressing a C-terminal Foxp3-AID fusion protein, detectable via the V5 epitope tag (Fig. 1B). The inclusion of an IRES-GFP moiety in the allele enables continued visualization of T_REG_ cells after Foxp3 protein degradation is complete, while the diphtheria toxin receptor (DTR) cassette enables inducible T_REG_ cell ablation (3). These mice were bred to *Rosa26*^*Tir1-F74G*^ mice, which constitutively express Tir1-F74G in all cells (30, 31). Hemizygous male *Foxp3*^*AID-V5-IRES-DTR-GFP/Y*^*Rosa26*^*Tir1-F74G/Tir1-F74G*^ and homozygous female *Foxp3*^*AID-V5-IRES-DTR-GFP/AID-V5-IRES-DTR-GFP*^*Rosa26*^*Tir1-F74G*/*Tir1-F74G*^ animals, henceforth referred to as *Foxp3*^*AID*^ mice, were healthy and showed no signs of spontaneous T cell activation, which could be effectively induced by diphtheria toxin (DT)-mediated T_REG_ cell depletion (Fig. S1A-C). GFP^+^ T_REG_ cells from *Foxp3*^*AID*^ mice were uniformly V5^+^ (Fig. 1C). To define the kinetics of Foxp3 degradation, we injected *Foxp3*^*AID*^ mice with 5-Ph-IAA at different timepoints prior to the isolation and analysis of splenic GFP^+^ T_REG_ cells (Fig. S1D-E). Using intracellular antibody staining for either the V5 tag or an endogenous Foxp3 epitope, we found that approximately 90-95% of Foxp3 protein was degraded 10 hours after 5-Ph-IAA injection (Fig. 1D). Foxp3 protein gradually recovered to pre-treatment levels after approximately 48 hours (Fig 1E). Thus, the Foxp3-AID fusion protein is correctly expressed, functional, and rapidly degraded upon *in vivo* administration of 5-Ph-IAA.

### Foxp3 protein is dispensable for short-term maintenance of steady-state T_REG_ cells

High levels of Foxp3 protein are essential to establish immunological tolerance. Previous work has shown that a constitutive reduction in Foxp3 protein levels of approximately 80% caused by an mRNA-destabilizing reporter gene insertion in the 3’ UTR of the mouse *Foxp3* gene was sufficient to induce aggressive early onset autoimmunity (32). Whether Foxp3 protein is continuously required to maintain T_REG_ cell identity and suppress T cell activation in steady-state adult animals is less clear. To address this question, we induced continuous Foxp3 protein depletion by injecting *Foxp3*^*AID*^ mice with 5-Ph-IAA every 48 hours for two weeks. To ensure protein degradation between injection timepoints, we also implanted a small osmotic pump continuously delivering a low dose of 5-Ph-IAA (Fig. 1F). GFP^+^ T_REG_ cells isolated 48 hours after the final 5-Ph-IAA injection showed a 55% reduction in Foxp3 protein levels compared to PBS-treated control animals (Fig. 1G). Thus, we achieved a continuous reduction in Foxp3 protein levels fluctuating between 95% depletion and 55% depletion, measured 10 hours and 48 hours post-injection, respectively (Fig. 1D, F, G).

This sustained reduction was associated with an increase in the frequencies of activated CD44^+^ GFP^-^ CD4 and CD8 T cells in the spleen and increased production of the pro-inflammatory cytokines IFN-γ, IL-4, and IL-17 (Fig. 1H-K). However, GFP^+^ CD4 T cell frequencies were not reduced and even slightly increased compared to PBS-treated controls (Fig. 1L). These cells showed reduced expression of the high-affinity interleukin-2 receptor (IL-2R) α-chain CD25 but maintained normal *Foxp3* gene transcription, as measured by GFP intensity. These results therefore suggested that continuously high levels of Foxp3 protein were dispensable for sustained *Foxp3* expression and T_REG_ cell maintenance under steady-state conditions, but required for suppressive function. However, when we directly compared the effects of Foxp3 protein depletion to DT-induced T_REG_ cell ablation, it became apparent that the latter caused much more dramatic effector CD4 and CD8 T cell expansion than the former (Fig. 1I, M, S1A-C). Thus, despite losing some of their activity, T_REG_ cells depleted of Foxp3 protein were still capable of suppressing T cell expansion to a considerable degree. Consistently, these cells also maintained their expression of various T_REG_ cell surface markers and suppressive molecules, including CD39, CD73, CTLA-4, GARP (LRRC32), and GITR (Fig. S1F-G). We therefore conclude that splenic T_REG_ cells were not only maintained but also continued to exert some of their suppressive functions following Foxp3 protein depletion under steady-state conditions.

**Fig. 2:**
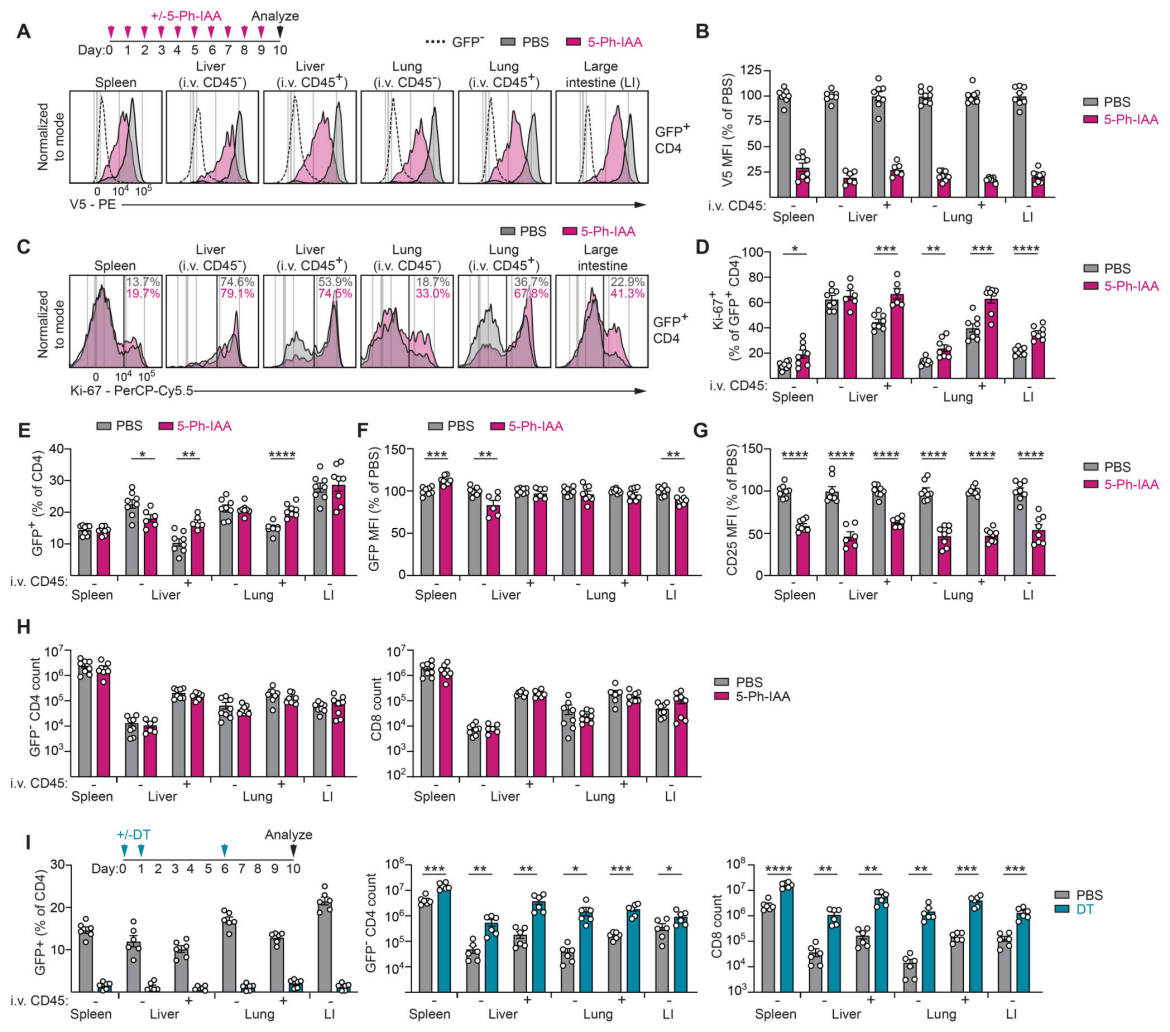
Proliferative non-lymphoid tissue T_REG_ cells persist following short-term Foxp3 depletion. (**A-B**) Male and female *Foxp3*^*AID*^ mice were injected with 5-Ph-IAA or PBS for 10 consecutive days. An eFluor450-labeled antibody against CD45 was injected intravenously (i.v.) 3 minutes before euthanizing the animals to distinguish vascular-associated (i.v. CD45^+^) and tissue-associated (i.v. CD45^-^) immune cells. Foxp3 (V5) protein levels were measured in GFP^+^ CD4 T cells from spleen, liver, lung, and large intestine lamina propria (LI). GFP^-^ CD4 T cells are shown as a negative control. (**C-D**) Flow cytometry data showing Ki-67 staining on GFP^+^ CD4 T cells. (**E**) Frequency of GFP^+^ cells among CD4 T cells in indicated organs. (**F**) GFP geometric mean fluorescence intensity (MFI) among GFP^+^ CD4 T cells. (**G**) CD25 levels on GFP^+^ T_REG_ cells, normalized to PBS-treated controls. (**H**) GFP^-^ CD4 and CD8 T cell counts across different organs after 10 days of continuous Foxp3 protein depletion. Panels A-H show pooled data from 2 independent experiments with a total of 6-8 mice per group. (**I**) *Foxp3*^*AID*^ mice were injected with diphtheria toxin (DT) on day 0, 1, and 6. GFP^+^ CD4 T cell frequencies and GFP^-^ CD4 and CD8 T cell counts analyzed on days 10-12. Pooled data from 2 independent experiments with a total of 6 mice per group. Error bars show mean with SEM. *P* values were calculated using unpaired two-tailed Student’s t-test. *: *P* ≤ 0.05, **: *P* ≤ 0.01, ***: *P* ≤ 0.001, ****: *P* ≤ 0.0001.

We hypothesized that it was the quiescent, non-proliferative state of splenic Treg cells that enabled them to tolerate the short-term depletion of their lineage-defining transcription factor. To examine this hypothesis, we analyzed the effects of Foxp3 protein degradation on non-lymphoid tissue T_REG_ cells, which depend on T cell receptor (TCR) stimulation for their differentiation and migration into organs and are much more proliferative than their splenic counterparts (33–35). To ensure more complete depletion of Foxp3 protein in these experiments, we administered 5-Ph-IAA every 24 hours for 10 consecutive days before analyzing the vascular (i.v. anti-CD45^+^) and extravascular (i.v. anti-CD45^-^) compartments of the liver, lungs, and large intestine (Fig 2A-B, S2A-B). Consistent with an established role for Foxp3 in maintaining anergy (9), T_REG_ cells across multiple different organs more highly expressed the proliferation marker Ki-67 in the absence of Foxp3 protein (Fig. 2C-D). However, although up to 70% of GFP^+^ non-lymphoid tissue T_REG_ cells were Ki-67^+^, these cells were largely maintained in the absence of Foxp3, with a slight decrease observed only in the extravascular compartment of the liver (Fig. 2E). GFP levels were also largely unaffected by Foxp3 protein depletion, with minor reductions observed in the extravascular compartment of the liver and the large intestine lamina propria (Fig. 2F). In contrast, CD25 levels on T_REG_ cells were uniformly reduced upon Foxp3 protein depletion (Fig. 2G, S2C). Thus, even highly proliferative non-lymphoid tissue T_REG_ cells could persist for at least 10 days in the absence of Foxp3.

To determine if these cells also maintained their suppressive activity, we compared the effects of 5-Ph-IAA-induced Foxp3 protein depletion and DT-induced T_REG_ cell ablation on GFP^-^ CD4 and CD8 T cell numbers. While the former had no significant effect (Fig. 2H), the latter induced dramatic T cell expansion in all organs (Fig. 2I). Thus, T_REG_ cells across different tissues of steady-state animals continued to suppress rampant T cell expansion for at least 10 days in the near-complete absence of their lineage-defining transcription factor, Foxp3.

### Foxp3 is essential for T_REG_ cells responding to type 1 inflammation

The results of our protein degradation experiments were in apparent contrast with a previous study that reported a continuous requirement for Foxp3 in maintaining the identity and function of mature T_REG_ cells (11). This study used retrovirally-expressed Cre recombinase to induce the deletion of a *lox*P-flanked *Foxp3* allele in mature T_REG_ cells before transferring these cells, together with conventional CD4 T cells, into lymphopenic recipients. T_REG_ cells in this experiment downregulated the expression of *Foxp3* and other T_REG_ cell signature genes and lost their suppressive activity, leading to the conclusion that T_REG_ cell maintenance is continuously dependent on Foxp3. We considered the possibility that this apparent discrepancy with our own results was attributable to the small amount of Foxp3 protein left after 5-Ph-IAA-induced protein degradation, compared to the complete absence of functional Foxp3 protein following the genetic deletion of *Foxp3*. Alternatively, because adoptively transferred T_REG_ cells become highly activated in response to the lymphopenic environment and pro-inflammatory cytokines produced by the co-transferred conventional CD4 T cells, they may be more strongly dependent on Foxp3 than their resting counterparts residing in healthy organs of unperturbed mice.

**Fig. 3:**
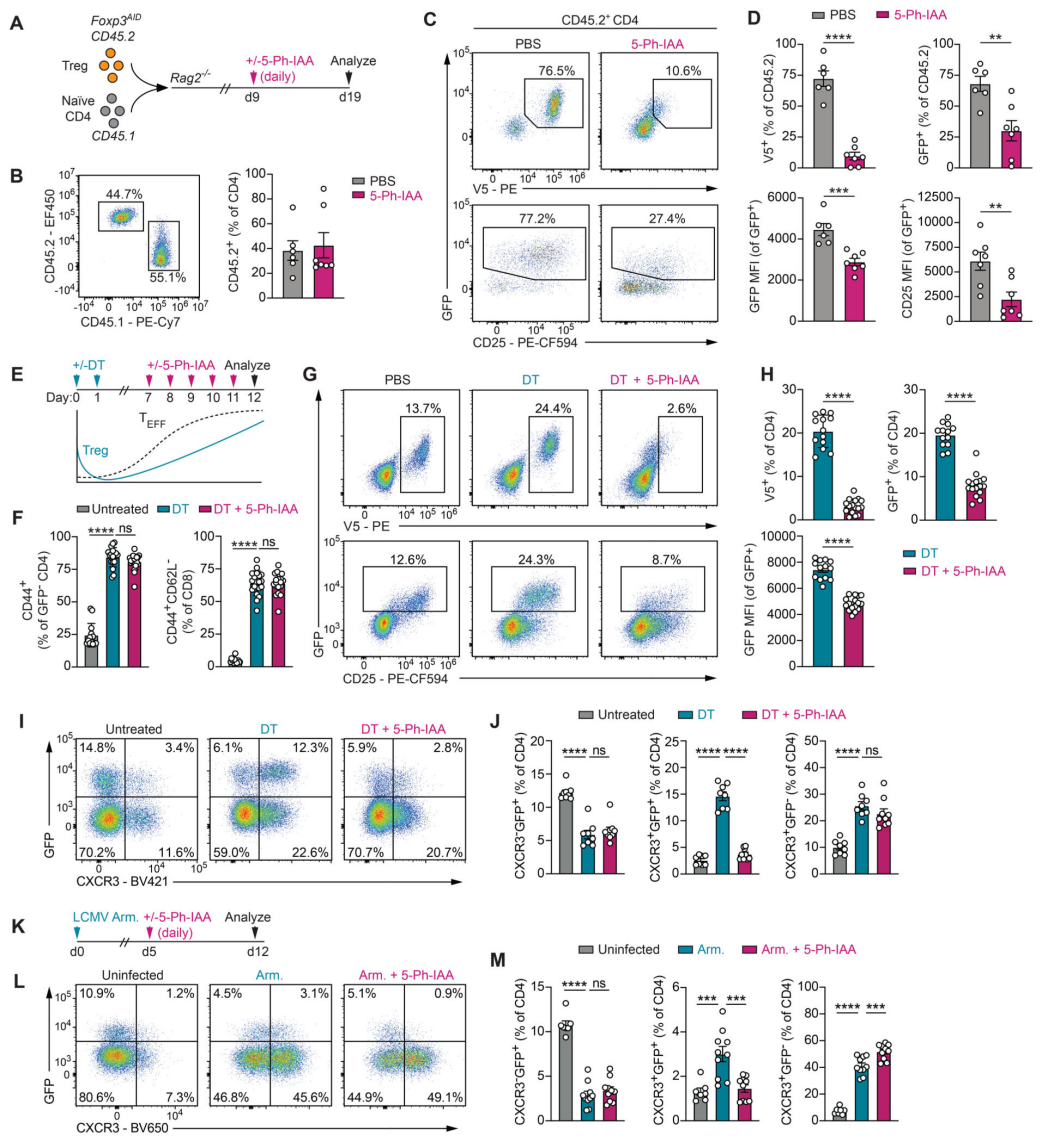
Acutely activated T_REG_ cells require Foxp3. (**A**) Experimental setup. (**B**) Representative CD45 staining on splenic CD4 T cells and summary data showing percentage of CD45.2^+^ CD4 T cells. (**C-D**) V5, GFP, and CD25 levels on splenic CD45.2^+^ CD4 T cell populations. Panels A-D show pooled data from two independent experiments with 6-7 mice per group. (**E**) Experimental setup. (**F**) Splenic effector CD4 and CD8 T cell frequencies on day 12. Pooled data from 4 independent experiments with 14-20 mice per group (**G-H**) GFP^+^ and V5^+^ cell frequencies among splenic CD4 T cells and GFP MFI on day 12. Panel H shows pooled data from three independent experiments with 13-16 mice per group. (**I-J**) GFP and CXCR3 staining on CD4 T cells on day 12. Pooled data from two independent experiments with a total of 8-9 mice per group. (**K**) Experimental setup. *Foxp3*^*AID*^ mice were infected with 2x10^5^ pfu LCMV Armstrong (LCMV Arm.). Foxp3 protein was depleted from day 5 onwards through daily injections of 5-Ph-IAA. (**L-M**) GFP and CXCR3 staining on splenic CD4 T cells on day 12 post-infection. Pooled data from two independent experiments with 7-10 mice per group. Error bars show mean with SEM. *P* values were calculated using unpaired two-tailed Student’s *t-*tests (D, H) or one-way ANOVA with Tukey’s multiple comparisons test (F, J, M). ns: *P* > 0.05, *: *P* ≤ 0.05, **: *P* ≤ 0.01, ***: *P* ≤ 0.001, ****: *P* ≤ 0.0001.

To distinguish between these possibilities, we transferred 3x10^5^ double-sorted (>99% pure) GFP^+^ T_REG_ cells isolated from *Foxp3*^*AID*^ mice together with equal numbers of congenically-marked naïve CD4 T cells into *Rag2*^*-/-*^ mice before inducing Foxp3 protein degradation (Fig. 3A). In PBS-treated animals, we found that approximately 75% of Foxp3-sufficient T_REG_ cells maintained GFP expression 19 days post-transfer (Fig. 3B-D). In contrast, and consistent with prior observations (11), depletion of Foxp3 protein from day 9 onwards led to a significant loss of GFP^+^ cells, with the remaining T_REG_ cells showing reduced *Foxp3* gene transcription and CD25 levels (Fig. 3B-D). Thus, Foxp3 protein degradation using the AID2 system could recapitulate a previously reported phenotype associated with inducible genetic *Foxp3* deficiency. Our results therefore suggested that not all T_REG_ cells are equally dependent on a continuously high level of Foxp3 protein and that a particularly strong requirement for Foxp3 may exist in T_REG_ cells exposed to inflammatory environments.

To determine if T_REG_ cells in other inflammatory settings were similarly sensitive to Foxp3 protein depletion, we first analyzed T_REG_ cells exposed to systemic autoimmune inflammation. The DT-induced depletion of DTR-expressing T_REG_ cells causes the rapid expansion and activation of self-reactive T_CON_ cells producing a variety of cytokines (5). Upon cessation of DT treatment, newly generated T_REG_ cells expanding in this inflammatory environment acquire transiently enhanced suppressive functions and dampen autoimmunity until homeostasis is restored (17, 36). To induce systemic autoimmunity, we injected *Foxp3*^*AID*^ mice with two consecutive daily doses of DT. Starting from day 7, when T_REG_ cell frequencies have already recovered to a considerable degree (3), we depleted Foxp3 protein by daily injection of 5-Ph-IAA (Fig. 3E). Analysis of splenic T cell populations on day 12 revealed dramatic expansion of CD44^+^ effector CD4 and CD8 T cells relative to healthy control animals (Fig. 3F). Foxp3 degradation under these conditions caused a reduction in the frequency of GFP^+^ T_REG_ cells, with the remaining population showing lower *Foxp3* gene transcription as measured by GFP levels (Fig. 3G-H). Thus, T_REG_ cells responding to DT-induced autoimmune inflammation were continuously dependent on Foxp3. The observation that most, but not all GFP^+^ cells were lost in this setting also raised the possibility that certain T_REG_ cell subpopulations were more strongly affected by Foxp3 protein depletion than others. A specialized T_REG_ cell subset expressing the transcription factor T-bet and the chemokine receptor CXCR3 has been shown to arise under conditions of type 1 inflammation in response to the cytokine IFN-#x03B3; (37–39). We found that these T_H_1-like T_REG_ cells, identified by either T-bet or CXCR3 staining, accounted for approximately 70% of T_REG_ cells responding to DT-induced autoimmune inflammation and that this subset was selectively lost following Foxp3 depletion (Fig. 3I-J, S3A-C).

We next asked if T_H_1-like T_REG_ cells generated during an acute viral infection were similarly dependent on Foxp3. To this end, we infected mice with the Armstrong strain of lymphocytic choriomeningitis virus (LCMV), which induces a potent T_H_1 and CD8 T cell response and the production of IFN-γ. As previously reported, approximately 50% of T_REG_ cells in the spleens of LCMV-infected mice expressed CXCR3 on day 12 post-infection (Fig. 3K-M) (40, 41). Depletion of Foxp3 protein from days 5 to 12 caused a selective loss of GFP^+^ CXCR3^+^ T_REG_ cells, consistent with the notion that Foxp3 is continuously required to sustain this subset of inflammation-induced T_REG_ cells. Thus, in contrast to resting T_REG_ cells residing in healthy organs of unperturbed mice, T_REG_ cells actively responding to autoimmune inflammation and viral infection are continuously dependent on high levels of Foxp3 protein.

### Immediate gene-regulatory functions of Foxp3

**Fig. 4:**
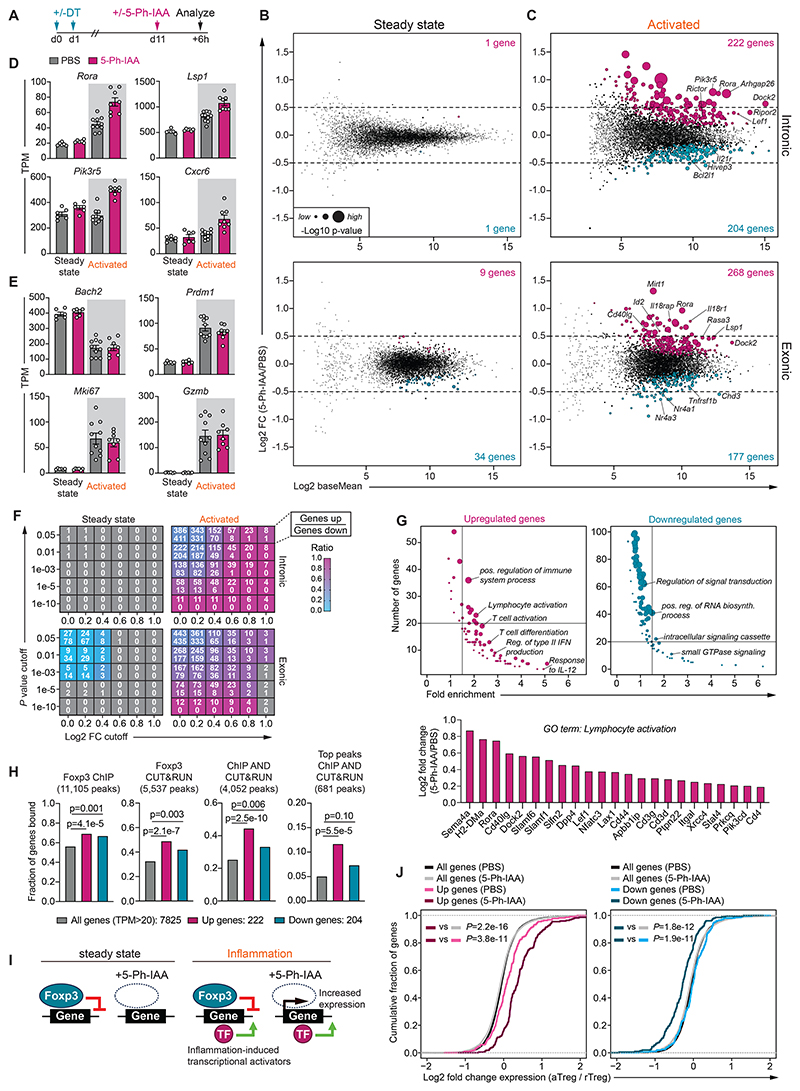
Immediate transcriptional functions of Foxp3 in activated T_REG_ cells (**A**) Experimental setup. (**B-C**) Differential gene expression analysis in steady-state (left) or activated (right) GFP^+^ CD4 T cells from pooled secondary lymphoid tissues of animals treated with 5-Ph-IAA or PBS for 6 hours. Analysis of intronic (top) and exonic (bottom) reads. Genes with adjusted *P* <0.01 are highlighted. RNA-sequencing samples were pooled from 7 independent sorts, with a total of 6-10 biological replicates per condition. (**D-E**) Expression of selected genes in transcripts per million (TPM). (**F**) Each cell shows the number of upregulated (top) and downregulated (bottom) genes at a given *P* value and fold change (FC) cutoff. Cells are colored according to the ratio of up- and downregulated genes. (**G**) Gene Ontology (GO) term enrichment analysis for differentially expressed (DE) genes (top). Each dot represents a GO term. Dot size reflects statistical significance of the enrichment. Upregulated genes associated with GO term lymphocyte activation (bottom). (**H**) DE genes identified in activated T_REG_ cells were linked to Foxp3 binding sites identified by ChIP-seq, CUT&RUN, the intersection of peaks identified by both techniques or the intersection of the top 2000 most highly bound sites identified by both techniques using public data from GSE154680. (**I**) Model for activation-dependent gene regulation by Foxp3. (**J**) Differential gene expression between activated T_REG_ (aTreg) cells and resting T_REG_ (rTreg) cells, measured in the presence (PBS) or absence (5-Ph-IAA) of Foxp3 protein. Analysis was performed for three gene sets: all expressed genes (all genes), genes upregulated after Foxp3 degradation in activated T_REG_ cells (Up genes) and genes downregulated after Foxp3 degradation in activated T_REG_ cells (Down genes). *P* values from hypergeometric test (H) or one-sided Kolmogorov-Smirnov test (**J**).

We next sought to define the immediate transcriptional targets of Foxp3 under steady-state and inflammatory conditions. To this end, we performed total RNA-seq analysis on GFP^+^ CD4 T cells isolated from pooled secondary lymphoid tissues 6 hours after injection of 5-Ph-IAA (Fig. 4A). To induce acute inflammation, animals were transiently treated with DT 11 days prior to Foxp3 protein depletion (Fig. 4A). Intronic reads from the resulting datasets were used as a proxy for nascent transcripts as previously described (42); however, because many genes lack introns or may be co-transcriptionally spliced, we also separately analyzed exonic reads. Acute Foxp3 protein depletion only minimally affected the transcriptome of steady-state T_REG_ cells (Fig. 4B). In contrast, we identified many differentially expressed genes in acutely activated T_REG_ cells, including transcription factors (e.g. *Lef1, Rora, Nr4a1, Nr4a3*, and *Id2*), signaling molecules (e.g. *Rasa3, Dock2, and Rictor*), and cell surface proteins (e.g. *Il18r1, Il21r*, and *Cd40lg*) (Fig. 4C-D, Data File S1). Transcriptional features characteristic of activated T_REG_ cells such as the downregulation of *Bach2* and upregulation of *Prdm1, Gzmb*, and the proliferation marker *Mki67* were unaffected by acute Foxp3 protein depletion (Fig. 4E).

Although the immediate transcriptional targets of Foxp3 included both repressed and activated genes, we found that repression was much more pronounced at various fold-change and *P* value cutoffs (Fig. 4F). Moreover, while genes upregulated after Foxp3 degradation were strongly enriched for genes involved in T cell activation and differentiation, genes downregulated upon Foxp3 depletion were not (Fig. 4G). Finally, we found that upregulated genes were more strongly enriched for Foxp3 binding, as measured by ChIP-seq and CUT&RUN analysis (19) (Fig. 4H). Together, these observations suggest that Foxp3 acts predominantly as a direct transcriptional repressor in activated T_REG_ cells.

While very few individual genes were significantly affected by Foxp3 degradation in steady-state T_REG_ cells, we found that these cells to a lesser degree also showed significant differential expression of the collective Foxp3-dependent gene set identified in activated T_REG_ cells (Fig. S4A). Moreover, when steady-state T_REG_ cells were re-analyzed independently of their activated counterparts, more statistically significant differential gene expression could be detected; however, the magnitude of these changes was minimal (Fig. S4B-D). Notable genes included *Tcf7, Lef1*, and *Foxp3* itself, which was slightly de-repressed upon Foxp3 protein depletion (Fig. S4D). We first considered the possibility that protein degradation was simply more efficient in activated versus steady-state T_REG_ cells. However, a time course analysis revealed only minor differences in Foxp3 degradation kinetics. The largest difference was observed 2-hours after 5-Ph-IAA injection, at which time activated T_REG_ cells contained approximately 25% less Foxp3 protein than resting T_REG_ cells (Fig. S4E). At the timepoint of the analysis, 6-hours after 5-Ph-IAA injection, no statistically significant difference in Foxp3 protein levels could be detected between steady-state and activated T_REG_ cells.

To further account for the potentially confounding effects of these small differences in degradation kinetics, we analyzed how the genes differentially expressed after acute Foxp3 protein degradation were affected by constitutive genetic *Foxp3* deficiency at different stages of T_REG_ cell development. Here, we took advantage of a previously generated dataset comparing wildtype T_REG_ to “reporter-null” cells isolated from female *Foxp3*^*GFPKO/WT*^ mice (19). These animals remain healthy due to random X-chromosome inactivation and expression of the wildtype *Foxp3* allele in half of their T_REG_ cells. Wildtype T_REG_ and GFPKO cells were compared at the CD62L^+^ “naïve” stage and the CD62L^-^ “effector” stage, induced by T cell receptor (TCR) stimulation (43) (Fig. S5A-C). Genes that were differentially expressed following protein degradation in activated T_REG_ cells were also differentially expressed in “effector” T_REG_ versus GFPKO cells, but not in their “naïve” counterparts (Fig. S5D-F). Thus, complete genetic *Foxp3* deficiency in “naïve” T_REG_ cells was insufficient to recapitulate the transcriptional changes resulting from acute Foxp3 protein degradation in activated T_REG_ cells. We therefore conclude that Foxp3 has *bona fide* activation-dependent gene-regulatory functions that are acutely required to maintain T_REG_ cell transcriptional identity under inflammatory conditions.

We considered several mechanisms through which an inflammation-dependent requirement for Foxp3 might arise. First, Foxp3 might bind to different genes under inflammatory conditions and alter the expression of these new targets (20). Second, Foxp3 may recruit activation-dependent co-factors whose expression or activity is induced under inflammatory conditions (17). Finally, Foxp3-dependent repression of its constitutively bound targets may counteract inflammation-induced transcriptional activators that are absent in steady-state T_REG_ cells (Fig. 4I). Arguing against the first model, we previously found that genome-wide Foxp3 binding patterns are largely unchanged under conditions of DT-induced autoimmune inflammation (17). Moreover, both the first and second models would predict that directly repressed Foxp3 target genes are downregulated in Foxp3-sufficient activated versus resting T_REG_ cells; however, we did not find this to be the case (Fig. 4J). Rather, expression of these genes was increased in activated Foxp3-depleted T_REG_ cells compared to all other conditions, which is most consistent with the third model (Fig. 4I, J). Thus, while other modes of regulation cannot be ruled out, our results suggest that the activation-dependent function of Foxp3 in maintaining T_REG_ cell transcriptional identity arises mostly from its ability to counteract inflammation-induced transcriptional activators.

### Cytokines dictate the effect of Foxp3 depletion

Because T_REG_ cells responding to autoimmune inflammation or infection *in vivo* are exposed to a complex mix of signals, we next sought to define the requirements for Foxp3 in T_REG_ cells responding to defined cytokine stimuli. The selective effects of Foxp3 protein depletion on the maintenance of T-bet^+^CXCR3^+^ T_REG_ cells suggested a role in modulating the response to T_H_1 polarizing cytokines. This was further supported by an enrichment for genes involved in the regulation of type II IFN production and response to interleukin-12 (IL-12) among the directly repressed transcriptional targets of Foxp3, including *Ifngr1, Jak2, Stat4*, and *Tbx21* (Fig. 4G, S6A-B). Moreover, using a previously published single-cell RNA-seq compendium of immune cell responses to 86 different cytokines (44), we found that the expression of genes directly repressed by Foxp3 was induced in T_CON_ cells responding to IL-12 administration (Fig. S6C). Thus, Foxp3 directly represses genes involved in T_H_1 responses.

**Fig. 5:**
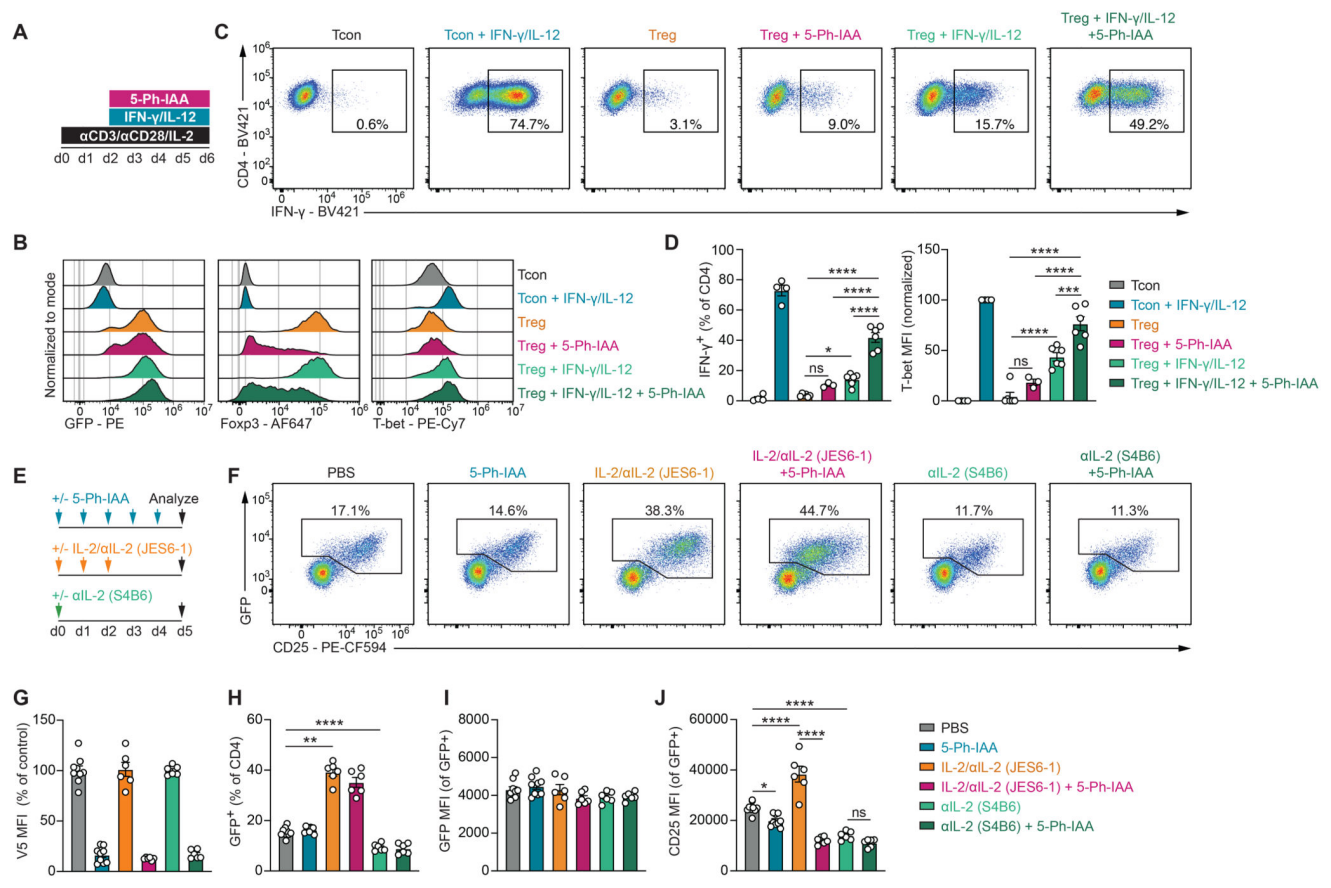
Cytokines dictate the effects of Foxp3 depletion. (**A**) Experimental setup. FACS-sorted GFP^+^ T_REG_ or GFP^-^ naïve T_CON_ CD4 T cells were isolated from pooled secondary lymphoid tissues of *Foxp3*^*AID*^ mice and activated *in vitro* on anti-CD3-coated plates with anti-CD28 and IL-2. 5-Ph-IAA and T_H_1-polarizing cytokines (IFN-γ and IL-12) were added to the cultures 48 hours post-activation, as indicated. (**B-D**) Flow cytometry analysis of GFP, Foxp3, T-bet, and IFN-γ levels. T_CON_ cells activated in the presence or absence of T_H_1-polarizing cytokines are gated on GFP^-^ CD4 T cells. T_REG_ cells are gated on GFP^+^ CD4 T cells. Cytokine production was measured after 3-hours of restimulation with PMA/Ionomycin/Brefeldin A/Monensin. T-bet levels are normalized to the positive control population (T_CON_ + IFN-γ/IL-12) after subtracting background fluorescence. Pooled data from 4 independent experiments with a total of 3-6 biological replicates per group. (**E**) Experimental design. (**F**) GFP and CD25 levels on splenic CD4 T cells of the indicated treatment groups. (**G-J**) Summary data showing Foxp3 (V5) and CD25 levels on splenic GFP^+^ CD4 T cells and the percentage of GFP^+^ cells among CD4 T cells. V5 levels are normalized to the average of their respective negative control groups that did not receive 5-Ph-IAA. Pooled data from three independent experiments with a total of 6-9 mice per group. Error bars show mean with SEM. *P* values were calculated using one-way ANOVA with Tukey’s multiple comparisons test. ns: *P* > 0.05, *: *P* ≤ 0.05, **: *P* ≤ 0.01, ***: *P* ≤ 0.001, ****: *P* ≤ 0.0001.

The development of naïve CD4 T cells into T_H_1 cells is initiated by TCR stimulation and IFN-γ-dependent activation of STAT1, which induces expression of the transcription factor T-bet and upregulation of the IL-12 receptor component IL-12Rβ2 (45, 46). IL-12-dependent activation of STAT4 then synergizes with T-bet to induce expression of the T_H_1 cell signature cytokine IFN-γ (47). T_REG_ cells are known to be less responsive to T_H_1 polarizing cytokines than T_CON_ cells (38). This is thought to prevent T_REG_ cells from producing IFN-γ, while still enabling the expression of T-bet and the downstream chemokine receptor CXCR3, necessary for migration to sites of type I inflammation. The role of Foxp3 in this “abortive” T_H_1 cell differentiation process is still unknown. We therefore investigated if Foxp3 was acutely required to limit IFN-γ production by T_REG_ cells stimulated under T_H_1 polarizing conditions *in vitro* (Fig. 5A). Foxp3-depleted T_REG_ cells activated in the presence of T_H_1 polarizing cytokines expressed higher levels of T-bet and more frequently produced IFN-γ than either Foxp3-sufficient T_REG_ cells activated under the same conditions or Foxp3-depleted T_REG_ cells activated in the absence of polarizing cytokines (Fig. 5B-D). Thus, Foxp3 directly represses multiple genes in the T_H_1 cell differentiation pathway and prevents IFN-γ production by T_REG_ cells upon exposure to type 1 inflammation.

Because of its central role in T_REG_ cell maintenance, we next analyzed how responsiveness to IL-2 was impacted by Foxp3 depletion. Both T_REG_ cells and activated T_CON_ cells rely on IL-2 as a critical growth factor to support their proliferation and survival (48). Naïve T cell activation via the TCR and co-stimulatory receptors triggers IL-2 production and expression of CD25, which pairs with the IL-2Rβ-chain and common γ-chain to form the high affinity IL-2 receptor (IL-2R). IL-2 then acts in an autocrine or paracrine manner to activate the IL-2R and downstream STAT5 signaling, which induces expression of genes associated with T cell proliferation and survival as well as further upregulation of CD25 (49). Because T_REG_ cells are incapable of producing IL-2, they rely solely on IL-2 produced by T_CON_ cells. Moreover, IL-2-induced STAT5 acts directly on regulatory elements in the *Foxp3* locus to maintain its transcriptional activity (50, 51). Finally, because T_REG_ cells express constitutively high levels of CD25, they can effectively outcompete T_CON_ cells for IL-2 and suppress their expansion (52, 53).

To define the direct gene-regulatory functions of Foxp3 in IL-2-stimulated T_REG_ cells, we injected mice with IL-2/anti-IL-2 (JES6-1) monoclonal antibody complexes that selectively engage and expand T_REG_, but not T_CON_ cells (54, 55), before acutely degrading Foxp3 protein for 6 hours (Fig. S7A). IL-2 stimulation induced transcriptional changes in approximately 2,500 genes, including those encoding the IL-2Rα- and β-chains and the anti-apoptotic protein Bcl-2 (Fig. S7B). Compared to T_REG_ cells responding to autoimmune inflammation, Foxp3 degradation in IL-2-stimulated T_REG_ cells affected a relatively small number of genes (Fig. S7C-D). Again, Foxp3 acted primarily as a transcriptional repressor. Among its directly regulated targets were *Pde3b*, whose downregulation contributes to the metabolic fitness of T_REG_ cells (9); *Tcf7*, encoding a chromatin remodeling transcription factor with widespread effects on the epigenetic landscape of T_REG_ and T_CON_ cells (19); *Themis*, a modulator of TCR signaling involved in thymocyte selection (56); and *Il7r*, another STAT5-activating cytokine receptor whose downregulation underlies the selective dependence of T_REG_ cells on IL-2. Finally, Foxp3 repressed *Cdk8*, encoding a negative regulator of STAT5 activity, inhibition of which is sufficient to induce T_REG_ cell differentiation (57) (Fig. S7E).

To test how Foxp3 depletion affected T_REG_ cell responsiveness to IL-2, we injected 5-Ph-IAA for 5 consecutive days in mice treated with either activating IL-2/anti-IL-2 (JES6-1) antibody complexes, or a distinct anti-IL-2 antibody (S4B6) that blocks the binding of IL-2 to CD25 (Fig. 5E). IL-2/anti-IL-2 complexes induced the expansion of GFP^+^ T_REG_ cells, while IL-2 neutralization induced their contraction, neither of which were significantly impacted by Foxp3 protein depletion (Fig. 5F-I). However, we found that the IL-2-induced upregulation of CD25 on T_REG_ cells was entirely Foxp3 dependent (Fig. 5J). Conversely, when IL-2 was neutralized, Foxp3 protein degradation no longer had any effect on CD25 levels. Importantly, T_REG_ cells continued to express higher levels of CD25 thanGFP^-^ T_CON_ cells even under conditions of IL-2 neutralization and Foxp3 protein depletion (Fig. 5F). This observation is consistent with previous work showing that *Foxp3*^*GFPKO*^ “reporter-null” cells express CD25 levels lower than wildtype T_REG_ cells, but higher than resting T_CON_ cells (9). Thus, while higher baseline expression of CD25 by T_REG_ cells is a Foxp3-independent feature, the upregulation of CD25 in response to IL-2 is Foxp3-dependent. We therefore conclude that Foxp3 regulates CD25 levels in an IL-2-dependent manner.

Based on our observations, we hypothesized that the Foxp3-dependent regulation of CD25 may be selectively important during an ongoing immune response to ensure that T_REG_ cells appropriately scale their CD25 levels and can continue to outcompete activated T_CON_ cells for IL-2. To test this, we analyzed the effects of Foxp3 protein depletion on CD25 expression by T_REG_ and T_CON_ cells in the context of autoimmune inflammation (Fig. S7I). The GFP^-^ CD4 and CD8 T cells that expanded after transient T_REG_ cell depletion more frequently expressed CD25 than their steady-state counterparts, which was further enhanced when Foxp3 protein was depleted for the final 5 days of the experiment (Fig. S7F-H). A comparison of CD25 levels between T_REG_ cells and CD25^+^ T_CON_ cells revealed that the former expressed higher levels of CD25 in steady-state or DT-treated animals (Fig. S7I-J). In contrast, in animals treated with DT and 5-Ph-IAA, T_CON_ cells expressed higher levels of CD25 than T_REG_ cells. These results are consistent with the notion that Foxp3-dependent feedback regulation of CD25 is critical to enable effective competition for IL-2 with activated T_CON_ cells (Fig. S7K).

Together, our data show that the effects of Foxp3 protein depletion are dictated by cytokine exposure. Foxp3-dependent suppression of IFN-γ production occurs only in the presence of T_H_1 polarizing cytokines, while Foxp3-dependent regulation of CD25 occurs when IL-2 is present. These two examples thus illustrate how an inflammation-dependent requirement for continuous Foxp3 activity might arise.

### Systemic Foxp3 protein depletion induces selective anti-tumor immune response

**Fig. 6:**
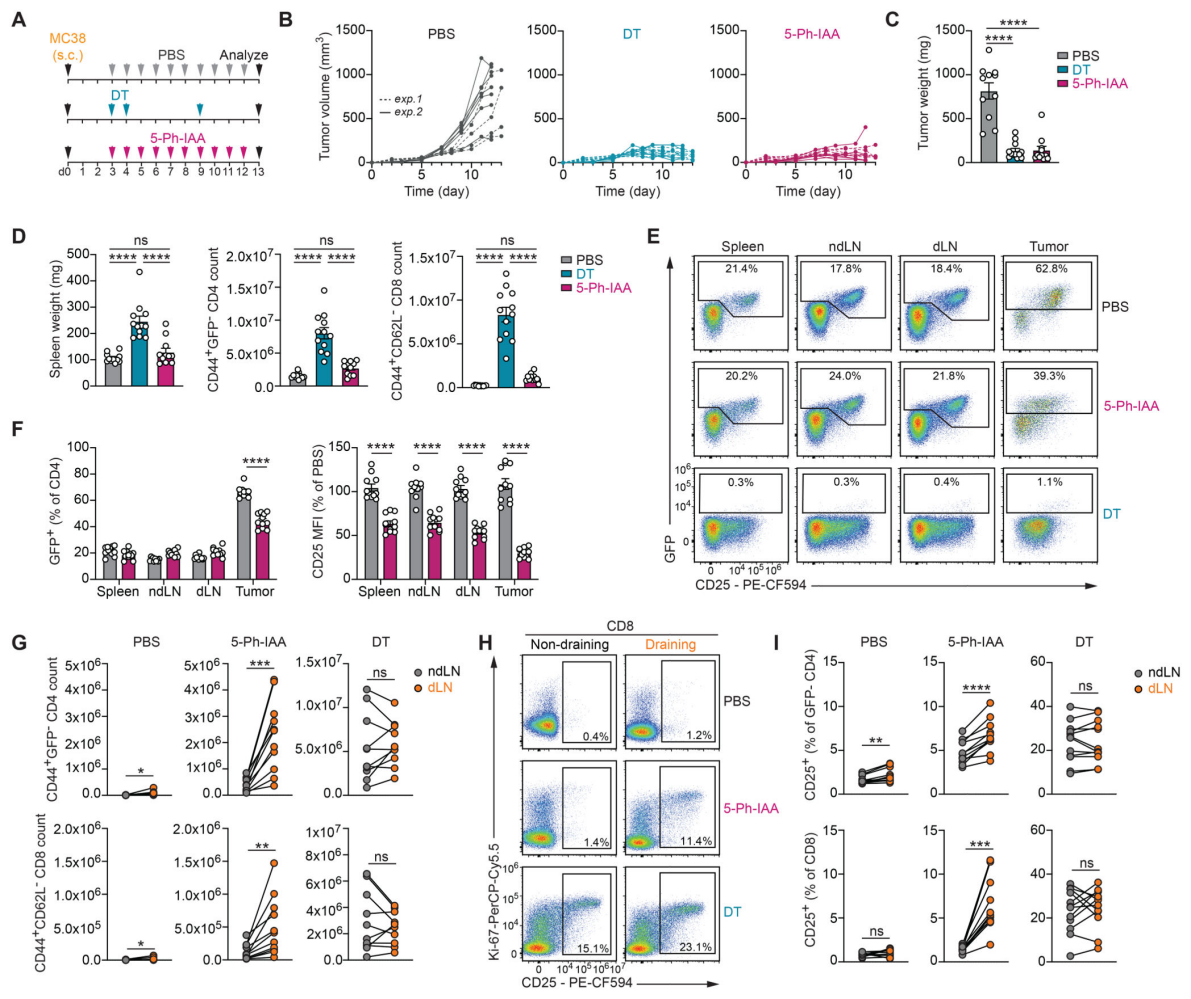
Short-term Foxp3 depletion drives localized anti-tumor immunity. **A)** Experimental setup. 1x10^6^ MC38 tumor cells were subcutaneously (s.c.) injected into the right flank of *Foxp3*^*AID*^ mice, which were subsequently treated with PBS, DT, or 5-Ph-IAA as indicated. Animals were analyzed on day 12 (experiment 2) or day 13 (experiment 1), when tumors from PBS-treated animals reached a critical size. **B)** Tumor volume measurements at indicated timepoints. **C)** Tumor weight at the end of the experiment. **D)** Spleen weight and effector T cell counts in the spleen at the end of the experiment. **E-F)** GFP and CD25 levels on CD4 T cells from the spleen, non-draining brachial and axillary lymph nodes (ndLN), draining brachial and axillary lymph nodes (dLN), or the tumor. **G)** Effector CD4 and CD8 T cell counts in the indicated organs. **H-I)** CD25 and Ki-67 staining on effector CD4 and CD8 T cells from the indicated organs. Pooled data from two independent experiments with 10-12 mice per experimental group. *P* values from one-way ANOVA with Tukey’s multiple comparisons test (C-D), or two-tailed unpaired (F) or paired (G, I) Student’s *t-*tests. ns: *P* > 0.05, *: *P* ≤ 0.05, **: *P* ≤ 0.01, ***: *P* ≤ 0.001, ****: *P* ≤ 0.0001.

Similar to T_REG_ cells exposed to other inflammatory settings, tumor-associated T_REG_ cells become highly activated in response to stimulatory cues in their microenvironment (17, 58). T_H_1-like T_REG_ cells accumulate in tumors and tumor-draining lymph nodes and interact with CD8 T cells and CXCL9-producing type 1 dendritic cells (DC1s) to suppress anti-tumor immunity through a variety of different mechanisms, including inhibition of antigen cross-presentation, downregulation of costimulatory molecules, and suppression of pro-inflammatory cytokine production (59–61). We found that nearly all T_REG_ cells infiltrating subcutaneously implanted MC38 colorectal tumors were T-bet^+^ (Fig. S8A-B). Based on our observations in the autoimmunity and viral infection models, we hypothesized that systemic depletion of Foxp3 protein would selectively interfere with these tumor-associated T_REG_ cells, while leaving T_REG_ cells elsewhere in the body relatively unaffected. A single intraperitoneal injection of 5-Ph-IAA was sufficient to degrade approximately 75% of Foxp3 protein in GFP^+^ tumor-infiltrating T_REG_ cells,15 hours post-injection (Fig. S9A). Daily treatments with 5-Ph-IAA initiated on day 3 after tumor implantation caused the expansion of tumor-infiltrating effector T cells (Fig. S9B-E) and effectively inhibited tumor growth to a similar degree as complete DT-induced T_REG_ cell ablation (Fig. 6A-C). Initiation of 5-Ph-IAA treatment on day 7 also inhibited the growth of established tumors and prolonged survival (Fig. S9F). Conversely, withdrawal from 5-Ph-IAA led to tumor re-growth within approximately one to two weeks (Fig. S9G). Thus, Foxp3 protein depletion was as effective as T_REG_ cell ablation at inhibiting tumor growth. However, while T_REG_ cell ablation induced splenomegaly and systemic T cell expansion, Foxp3 protein depletion did not (Fig. 6D, 2H-I). This was consistent with a selective reduction in GFP^+^ T_REG_ cell frequencies in tumors, but not secondary lymphoid tissues of 5-Ph-IAA-treated mice (Fig. 6E-F, S9H). CD25 levels were reduced on T_REG_ cells across all tissues, but most dramatically within the tumor. Thus, Foxp3 protein depletion selectively affects highly activated tumor-infiltrating T_REG_ cells, while leaving T_REG_ cells in healthy organs relatively unperturbed.

To confirm the localized effect of Foxp3 protein depletion, we next compared the tumor-draining and non-draining lymph nodes of mice within the same treatment groups (Fig. 6G). Tumor-bearing animals treated with PBS showed low CD4 and CD8 effector T cell counts in both the draining and non-draining lymph nodes, while T_REG_ cell-depleted mice showed dramatic, but non-specific T cell expansion at both sites. In contrast, Foxp3 protein depletion caused a preferential expansion of effector CD4 and CD8 T cells in the tumor-draining, but not the non-draining lymph node. Further analysis of these cells revealed

an expanded population of proliferative Ki-67^+^CD25^+^ CD8 T cells in the tumor-draining lymph node of 5-Ph-IAA-treated mice that was largely absent from non-draining lymph nodes, or from PBS-treated controls (Fig. 6H-I). Thus, short-term systemic depletion of Foxp3 protein induces a localized anti-tumor immune response without causing significant T cell expansion or loss of GFP^+^ T_REG_ cells elsewhere in the body. Together, our results identify an inflammation-dependent requirement for continuous Foxp3 activity that can be exploited to selectively target tumor-infiltrating T_REG_ cells.

## Discussion

Despite extensive characterization of *Foxp3*-deficient mice under various conditions, the functional requirements for Foxp3 in mature T_REG_ cells have remained poorly defined. Using the AID2 system in combination with a bicistronic *Foxp3* reporter, we found that high levels of Foxp3 protein were largely dispensable for the short-term maintenance of *Foxp3*-expressing cells under steady-state conditions. Foxp3-depleted T_REG_ cells also continued to restrain T_CON_ cell expansion to a considerable degree. Conversely, T_REG_ cells that were activated in response to autoimmune-inflammation, viral infection, cancer, or adoptive transfer into lymphopenic hosts were acutely dependent on Foxp3 for their maintenance. Specifically, a subset of T-bet^+^CXCR3^+^ T_REG_ cells that arises in settings of type 1 inflammation was lost following Foxp3 protein depletion. Because T_REG_ cells that died or shut down *Foxp3* transcription can no longer be identified using our model, the exact fate of these cells is still unclear.

Our observations also shed new light on the direct gene regulatory functions of Foxp3, which in the past have been difficult to distinguish from indirect secondary consequences of constitutive *Foxp3* deficiency. We found that Foxp3 controls gene expression in an activation-dependent manner and acts predominantly as a transcriptional repressor by counteracting inflammation-induced transcriptional activators. Although not directly supported by our data, other activation-dependent modes of gene regulation, such as re-localization of Foxp3 to different target genes or recruitment of inflammation-induced co-factors may also contribute (17, 20). Importantly, because some of the proteins encoded by Foxp3-regulated genes can still undergo a degree of turnover within the 6-hour 5-Ph-IAA treatment period, we cannot fully exclude the possibility that minor secondary transcriptional changes occur even within this short time frame (62). While our data suggest that Foxp3 acts predominantly as a transcriptional repressor, a direct role for Foxp3 in activating gene expression cannot be ruled out. Moreover, certain modes of gene repression, such as DNA methylation or polycomb-mediated silencing may take more than 6 hours to reverse and could therefore be missed in our analysis. Among the genes acutely repressed by Foxp3 were *Tcf7*, a chromatin-remodeling transcription factor whose downregulation contributes indirectly to shaping the T_REG_ cell accessible chromatin landscape (19); *Cdk8*, a negative regulator of STAT5 activity, inhibition of which is sufficient to induce T_REG_ cell differentiation (57); the mammalian target of rapamycin (mTOR) complex 2 (mTORC2) adapter protein *Rictor*, genetic deletion of which can partially rescue the fitness and suppressive function of Foxp3 deficient “reporter-null” cells (63); and the transcriptional regulator *Id2*, overexpression of which is sufficient to cause loss of *Foxp3* expression under inflammatory conditions (64). Thus, inhibition or genetic deletion of genes directly repressed by Foxp3 can induce T_REG_ cell differentiation or partially rescue *Foxp3* deficiency, while their overexpression can drive T_REG_ cell instability.

Our work suggests that Foxp3 controls distinct facets of T_REG_ cell biology depending on exposure to specific external stimuli. Foxp3 directly repressed genes involved in the regulation of IFN-γ production and the response to IL-12. Accordingly, Foxp3-depleted T_REG_ cells exposed to T_H_1-polarizing cytokines *in vitro* expressed increased levels of T-bet and began to produce IFN-γ. Another cytokine that modulated the activity of Foxp3 was IL-2. We found that the Foxp3-dependent regulation of CD25 was induced by exposure to IL-2 and abrogated upon IL-2 neutralization. Thus, the effects of Foxp3 protein depletion are determined by the cytokine environment. It is likely that exposure to additional signals such as TCR stimulation, alarmins, glucocorticoids, prostaglandins, and various cytokines and chemokines influence the activity and cellular requirements for Foxp3 in distinct ways. We speculate that the loss of Foxp3-depleted Treg cells in settings of autoimmune inflammation, viral infection, or cancer may result from their exposure to a complex combination of these stimuli.

While systemic Foxp3 degradation might have been expected to cause catastrophic autoimmunity, we find that it rather specifically augments anti-tumor T cell responses. This effect was associated with a selective loss of GFP^+^ T_REG_ cells from tumors, but not from healthy organs. It is possible that prolonged depletion of Foxp3 protein for multiple weeks or months will eventually cause more severe autoimmunity; however, this is likely to be reversible when *Foxp3*-transcribing cells are maintained. In support of this notion, a previous report demonstrated that the restoration of Foxp3 protein levels in mice expressing a *Foxp3* reporter-null allele could effectively rescue T_REG_ cell suppressive activity and cure a pre-existing autoimmune phenotype (65). Thus, transient Foxp3 protein depletion in adult animals is unlikely to have long-term deleterious consequences.

Consistent with the relatively modest effects of Foxp3 protein depletion in steady-state adult mice, human T_REG_ cells subject to acute genetic *FOXP3* deletion were shown to retain a degree of *in vitro* suppressive activity for at least two weeks (66). Together, these observations raise the question as to why constitutive genetic *Foxp3* deficiency causes such severe and early-onset disease. Based on the results of our adoptive transfer experiments, we speculate that one factor may be that a strict requirement for Foxp3 exists in neonates, when the first cohorts of newly generated T cells undergo a phase of lymphopenia-driven activation (67). Together, our findings shed new light on the cellular and molecular functions of Foxp3 and provide a rationale for direct therapeutic targeting of Foxp3 in cancer.

## Materials and Methods

### Study design

The goal of the study was to understand the continuous requirement for high levels of Foxp3 protein in T_REG_ cells exposed to type 1 inflammation, in settings of autoimmunity, viral infection, and cancer. To this end, we developed a *Foxp3*^*AID-V5-IRES-DTR-GFP*^ allele that enables acute Foxp3 protein depletion, visualization and isolation of Foxp3-depleted T_REG_ cells, and inducible ablation of *Foxp3*-expressing cells. The effects of Foxp3 protein depletion under various conditions were analyzed using flow cytometry and RNA-sequencing.

### Mice

Animals were housed at the IMP/IMBA shared animal facility under standard specific pathogen-free (SPF) conditions at a room temperature of 22 °C and 55% humidity on a 14-hour light/10-hour dark cycle and unrestricted access to food and water. Mice were euthanized by carbon dioxide inhalation or cervical dislocation. All experimental and control animals were age-matched and included both male and female mice between 6-14 weeks of age. All animal experiments were carried out under valid project licenses approved by the Austrian Veterinary Authorities.

The *Foxp3*^*AID-V5-IRES-DTR-GFP*^ (*Foxp3*^*AID*^) and *Rosa26*^*Tir1-F74G*^ alleles were generated by CRISPR/Cas9-mediated genome editing in zygotes using the EASI-CRISPR approach (68). To generate the *Foxp3*^*AID*^ allele, a single-stranded DNA (ssDNA) repair template encoding a glycine-serine linker, the minimal AID polypeptide (69), and a V5 epitope tag, flanked by ∼100 bp sequences homologous to the genomic regions immediately upstream and downstream of the *Foxp3* stop codon was obtained from integrated DNA technologies (IDT). The repair template was injected together with recombinant Cas9 protein (IDT) and an sgRNA overlapping the *Foxp3* stop codon (IDT) into *Foxp3*^*IRES-DTR-GFP*^ mouse zygotes (3). Correct targeting was confirmed by Sanger sequencing PCR products amplified using primers outside of the 3’ and 5’ homology arms. The *Rosa26*^*Tir1-F74G*^ allele was generated by injecting a 200 bp repair template together with Cas9 protein and a sgRNA targeting codon 73 of Tir1 into *Rosa26*^*Tir1/Tir1*^ zygotes (30, 31). *Rag2*^*tm1Fwa*^ (*Rag2*^*-/-*^) mice and *Ptprc*^*a*^ (CD45.1) mice were bred in-house.

### Diphtheria toxin, 5-Ph-IAA, and antibody administration

DT was obtained from List Biological Laboratories (cat #150) and dissolved in PBS. Animals were injected intraperitoneally with 40μg/kg per dose. 5-Phenol-indole-3-acetic acid (5-Ph-IAA) was purchased from BioAcademia (cat #30-003-10), dissolved in PBS and injected at 0.3mg per dose via intraperitoneal injection. For continuous 5-Ph-IAA administration, 200μl Alzet osmotic pumps (cat #2002) were filled with 5-Ph-IAA or PBS solution according to manufacturer’s instructions and implanted subcutaneously. IL-2/anti-IL-2 treatments were performed as previously described (55). Briefly, 1μg recombinant IL-2 (Pepro Tech, 212-12-250UG) was pre-incubated with 5μg JES6-1 antibody (BioXcell, BE0043) for 30 min at 37°C and intraperitoneally injected into mice on three consecutive days before analysis on day five. For RNA-seq analysis of IL-2-expanded T_REG_ cells, mice were injected with IL-2/anti-IL-2 complexes on days 0 to 3. Mice were injected again with IL-2/anti-IL-2 complexes, with or without addition of 5-Ph-IAA, 6-hours before analysis on day 5. To discriminate vascular from tissue lymphocytes, we intravenously injected 2 μg of anti-CD45.2 (104, Invitrogen, 48-0454-82) in 150 μL of PBS three minutes before tissue harvesting, as previously described (70).

### Antibodies

The following antibodies were used for flow cytometry, with clones, venders, catalog numbers and dilutions as indicated: anti-CD25 (PC61, BD, 562694, 1:400), anti-CD39 (Duha59, BioLegend, 143812, 1:400), anti-CD4 (GK1.5, BioLegend, 100449, 1:400), anti-CD44 (IM7, Thermo Fisher Scientific, 45-0441-82, 1:400), anti-CD45.1 (A20, Cytek Biosciences, 60-0453-U100, 1:400), anti-CD45.2 (104, Invitrogen, 48-0454-82, 1:400), anti-CD5 (53-7.3, BD Biosciences, 612809, 1:400), anti-CD62L (MEL-14, BioLegend, 104445, 1:400), anti-CD73 (TY/11.8, BioLegend, 127206, 1:400), anti-CD8α (53-6.7, BioLegend, 100744, 1:400), anti-CD90.2 (30-H12, Cytek Biosciences, 60-0903-U100, 1:1000), Anti-CXCR3/CD183 (CXCR3-173, Biolegend, 126521, 1:200), anti-CTLA-4 (UC10-4B9, BioLegend, 106316, 1:200), anti-FOXP3 (150D/E4, BioLegend, B320014, 1:200), anti-GARP (F011-5, BioLegend, 142904, 1:400), anti-GITR (DTA-1, BioLegend, 126312, 1:400), anti-IFN-γ (50-7311, Cytek Biosciences, 50-7311-U100, 1:400), anti-IL-4 BV421 (11B11, BioLegend, 504127, 1:400), anti-T-bet (4B10, Biolegend, 644824, 1:400), anti-TCRβ (H57-597, BioLegend, 109249), Anti-TNF-α (MP6-XT22, Biolegend, 506314, 1:800), anti-V5 (TCM5, Thermo Fisher Scientific, 12-6796-42, 1:400).

### Lymphocyte isolation

For cell isolation from spleens, lymph nodes and livers, organs were collected in ice-cold FACS buffer (PBS with 2mM EDTA and 1% fetal calf serum) and meshed through a 100μm strainer (Corning, cat# 07-201-432) using the back of a syringe plunger. Erythrocytes were lysed using ACK lysis buffer (Thermo Fisher Scientific, cat# A1049201).

For isolation of immune cells from intestinal tissues, organs were incubated with DTT/EDTA buffer (PBS with 2mM L-glutamine, 10mM HEPES, 1x penicillin/streptomycin, 5% FCS, 1mM DTT and 2mM EDTA) for 20 minutes at 37°C shaking at 250 RPM. The intraepithelial lymphocytes released in this step were discarded before enzymatically digesting the remaining tissue with Collagenase/DNase buffer (RPMI-1640 with 2mM L-glutamine, 10mM HEPES, 1x penicillin/streptomycin, 5% FCS, 1 mg/mL collagenase A from Clostridium histolyticum (Sigma-Aldrich, cat #11088793001), and 1 μg/mL DNaseI (Sigma-Aldrich: Cat #10104159001)) for 25 minutes at 37°C shaking at 250 RPM in the presence of five 0.25-inch ceramic beads (MP Biomedicals, cat #116540412). Lamina propria lymphocytes released in this step were filtered through a 100μm strainer and washed in FACS buffer. Immune cells from lungs and tumors were isolated by digesting finely minced tissues with Collagenase/DNase buffer for 25 minutes at 37°C shaking at 250 RPM in the presence of five ceramic beads. Tumors were digested in a similar manner using digestion buffer containing 0.125U/mL collagenase D (Roche cat #11088858001) instead of collagenase A. Digested samples were filtered through a 100μm strainer and washed in FACS buffer. Liver and tumor samples were enriched for lymphocytes by centrifugation in 40% (vol/vol) PBS-adjusted Percoll solution (Thermo Fisher, cat #45–001-747).

### Cell Purification and flow cytometry

Single cell suspensions were prepared in ice-cold FACS buffer, washed once in PBS, and stained with Ghost Dye™ Red 780 (Cytek Biosciences, cat #13-0865-T500) for 10 minutes at 4°C to label dead cells. Cell surface antigens were stained for 20 minutes at 4°C using a mix of fluorophore-conjugated antibodies diluted in FACS buffer. Unfixed cells were analyzed immediately or fixed and permeabilized using the eBioscience Foxp3/Transcription Factor staining buffer set (ThermoFisher, cat #00-5523-00), before intracellular staining according to the manufacturer’s instructions. For intracellular cytokine measurements, cells were re-stimulated in vitro with 50ng/mL PMA (Sigma-Aldrich, P8139-1MG), 1μg/mL ionomycin (Sigma-Aldrich, I0634), 1μg/mL brefeldin A (Sigma-Aldrich, B6542-5MG) and 2μM monensin (Sigma-Aldrich, M5273-1G) in complete RPMI for 3 hours at 37°C before fixation and permeabilization (BD, Cytofix/Cytoperm kit, 554714) and antibody staining. Cells were washed and resuspended in FACS buffer and filtered through a 100-μm nylon mesh before analysis on a Cytek Aurora spectral flow cytometer. For T_REG_ cell sorting, pooled spleen and lymph node cells were enriched for CD4 T cells using the Mojosort™ Mouse CD4 T Cell Isolation Kit (BioLegend cat # 480033), stained with antibody cocktails as described, and sorted on BD Aria II.

### T cell transfer

3x10^5^ FACS-purified (>99% pure) GFP^+^ T_REG_ cells from pooled secondary lymphoid tissues of *Foxp3*^*AID*^ mice were mixed with an equal number of FACS-purified naïve CD44^-^CD62L^+^CD25^-^CD4^+^ T cells isolated from *Ptprc*^*a*^ (CD45.1) mice and retroorbitally injected into *Rag2*^*-/-*^ mice. Starting on day 9 post-transfer, animals received daily injections of 5-Ph-IAA before analysis on day 19.

### LCMV infection

Mice were infected intraperitoneally with 2x10^5^ pfu LCMV Armstrong. Starting on day 5 post-infection, the animals were injected daily with 0.3 mg of 5-Ph-IAA for a period of 7 consecutive days before analysis on day 12.

### Subcutaneous tumor implantation

10^6^ MC38 cells in PBS were mixed with an equal volume of Matrigel and injected subcutaneously into the right flank. Mice were treated with 5-Ph-IAA, DT, or PBS as described. Tumor size was measured every 2-3 days using calipers. Tumor volume (V) was calculated as V = 0.5 x length x width^2^.

### In vitro Treg cell polarization and T_H_1 cell differentiation

T_REG_ cells were expanded *in vivo* using IL-2/anti-IL-2 complexes, as previously described. Pooled spleen and lymph node cells were enriched for CD4 T cells using the Mojosort™ Mouse CD4 T Cell Isolation Kit (BioLegend cat # 480033). GFP^+^ T_REG_ cells and GFP^-^ CD44^lo^CD62L^hi^ naïve CD4 T cells were isolated by flow cytometry. 3x10^5^ GFP^+^ Treg cells were cultured in 24-well plates coated with 10 µg/mL anti-CD3 (clone 145-2C11, Bio X Cell cat # BE0001-1) and 10 µg/mL anti-CD28 (clone 37.51, Bio X Cell cat # BE0015-1) in complete RPMI supplemented with 2000U/mL IL-2 (Peprotech cat # 212-12). Cells were cultured for 2 days before adding fresh media supplemented with either 10 µg/mL anti-IFNγ (clone XMG1.2, BD Biosciences cat # 554409) and IL-2, or 67 ng/mL IFN-γ (Biolegend cat # 575306), 67 ng/mL IL-12 (R&D systems cat # 419-ML/CF) and IL-2. Foxp3 protein was degraded by adding 0.5 µg/mL 5-Ph-IAA to the culture media. For T_H_1 cell differentiation, 500k naïve CD4 T cells were activated under the same conditions as the T_REG_ cells but immediately supplemented with 100 ng/mL IFN-γ, 100 ng/mL IL-12 and 2000 U/mL IL-2. Cells were analyzed on day 6.

### RNA-sequencing

GFP^+^ T_REG_ cells were FACS purified from pooled spleen and lymph nodes of *Foxp3*^*AID*^ mice. RNA was extracted using an in-house kit. Briefly, sorted cells were lysed with a buffer containing guanidine thiocyanate, Triton X-100 and *β*-mercapthoethanol. RNA was captured using magnetic beads. Following a series of wash steps, the beads were incubated with a DNAse I-containing buffer (NEB, cat #M0303) for 15 minutes at 37°C to degrade genomic DNA. After washing, the RNA was eluted in 20uL nuclease-free water for 5 mins at 60°C from pre-dried beads. RNA quality and concentration were assessed using the Agilent DNF-471 RNA kit and Agilent Fragment Analyzer. Sequencing libraries were prepared using the SMARTer Stranded Total Pico Input RNA Kit v3 (Takara cat. #634487) according to the manufacturer protocol, with 10ng of input RNA. Sequencing was performed using NovaSeqX 10B XP flowcell, using paired-end reads with a length of 150bp.

### Bioinformatic analysis of RNA-sequencing data

After DNA sequencing, reads were trimmed using Cutadapt and filtered using standard illumina quality metrics. Reads from ribosomal DNA were removed using Bowtie 2. Remaining reads were aligned to the mm10 genome version of December 2011 (GRCm38) using STAR version 2.4.2a. To quantify reads aligning to exons and introns, we used a custom-curated and extended version of mm10 RefSeq (downloaded on December 6, 2018) in which intron annotations were created by extrapolating from the annotated exons of each transcript. Read quantification was performed separately for exons and introns over the whole gene body using subread featureCounts version 2.0.2. Reads were separated into two groups: those entirely overlapping with exons (exonic reads) and those overlapping entirely with introns or with intron-exon junctions (intronic reads). Raw counts were converted to normalized values calculated as transcripts per million (TPM) using concatenated exon and intron lengths. For differential gene expression analysis, read counts from all samples were normalized together and pairwise differential expression between 5-Ph-IAA-treated and control groups was analyzed using DESeq2 version 1.34.0 in R-4.1.2 (https://www.r-project.org). Two versions of this analysis were performed: 1) steady-state and activated T_REG_ cells analyzed together, or 2) steady-state and activated T_REG_ cells analyzed separately. False Discovery Rate (FDR) correction for multiple testing took into account the total number of gene-level tests carried out across all the pairwise comparisons. Numbers of differentially expressed genes were calculated at various adjusted *P* value and fold-change cutoffs among genes with TPM>20 in at least one condition. Enrichment for Foxp3 binding at differentially expressed genes was analyzed using publicly available ChIP-seq and CUT&RUN data (GSE154680). Foxp3 binding sites identified by ChIP-seq, CUT&RUN, the intersection of peaks identified by both techniques, or the intersection of the top 2000 most highly bound sites identified by both techniques were linked to the nearest gene. Enrichment for Foxp3 binding to different gene sets was determined using a hypergeometric test.

### Statistical analysis

Statistical analysis was performed using Prism (GraphPad, v10.4.1). Statistical tests for individual experiments are reported in the figure legends. Briefly, two-tailed paired or unpaired *t* tests were used to compare two groups of samples. Comparisons between multiple groups were performed using one-way or two-way analysis of variance (ANOVA), followed by post hoc Tukey or Šidák multiple comparisons tests. A log-rank test was used to compare survival curves.

## Supplementary Material

Supplementary materials

## Data Availability

The RNA sequencing data generated in this study are available through the Gene Expression Omnibus (GEO) database under accession number GSE289371. Tabulated data underlying the figures is provided in data file S2. All other data needed to support the conclusions of the paper are present in the paper or the Supplementary Materials. Custom code for intronic RNA-seq data analysis is available from https://github.com/Jaritz/jaeger_2025. The *Foxp3*^*AID-V5-IRES-DTR-GFP*^ mice will be shared upon request after completion of a material transfer agreement (MTA) with the Research Institute of Molecular Pathology.

## One-sentence summary

Acute Foxp3 protein depletion selectively affects Treg cells responding to type 1 inflammation.
