# Supplementary material for: Inducible protein degradation reveals inflammation-dependent function of the Treg cell lineage-defining transcription factor Foxp3
Source: Sci Immunol. Author manuscript; Available in PMC 2025 Jun 20. (PMC7617780; doi:10.1126/sciimmunol.adr7057)
Supplement: Supplementary materials [file EMS206340-supplement-Supplementary_materials.pdf]

Science Immunology
AAAS

Supplementary Materials for

Inducible protein degradation reveals inflammation-dependent function of the Treg lineage-defining transcription factor FOXP3

Christina Jäger[1,2], Polina Dimitrova[1,2], Qiong Sun[1], Jesse Tennebroek[1], Elisa Marchiori[1,2], Markus Jaritz[1], Rene Rauschmeier[1], Guillem Estivill[1,2], Anna Obenauf[1], Meinrad Busslinger[1], Joris van der Veeken[1#].

Corresponding author: joris.van.der.veeken@imp.ac.at

The PDF file includes:

Figs. S1 to S9

Other Supplementary Materials for this manuscript include the following:

Data File S1. Processed RNA-sequencing data.
Data File S2. Raw data file.
Data File S3. Oligonucleotide sequences.

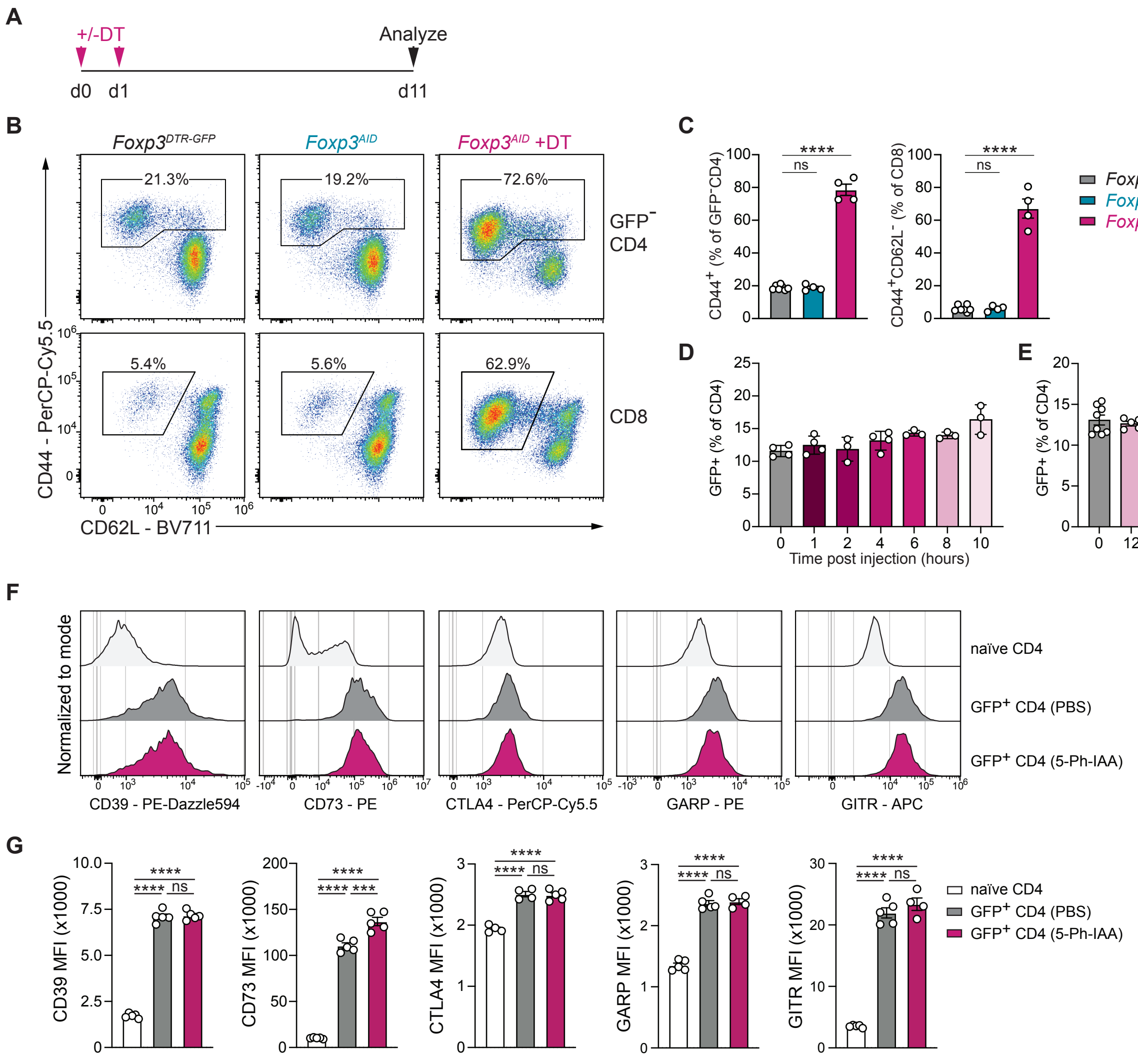

Fig. S1. Characterization of *Foxp3^{AID}* mice.

(**A**) Experimental design. Mice were intraperitoneally injected with diphtheria toxin (DT) and analyzed 11 days later. **(B-C)** Representative flow cytometry plots and summary data showing splenic effector T cell frequencies in 6-8 weeks old male *Foxp3$^{DTR-GFP/Y}$* and *Foxp3$^{AID-V5-IRES-DTR-GFP/Y}$* mice left untreated or treated with two consecutive daily doses of DT 11 days prior to analysis. Pooled data from two independent experiments with a total of 4-7 mice per group. **(D-E)** GFP^{+} cell frequencies among splenic CD4 T cells at the indicated timepoints after 5-Ph-IAA administration. Pooled data from 2 (D) or 3 (E) independent experiments with a total of 3-8 mice per timepoint. (**F-G**) Expression of T$_{REG}$ cell markers and suppressive molecules by naïve CD4 T cells and GFP^{+} T$_{REG}$ cells isolated from animals treated for 14 days with either PBS or 5-Ph-IAA, as described in Fig. 1F. Representative data from one of two independent experiments with 4-5 mice per group. Error bars show mean with SEM. *P* values were calculated using one-way ANOVA with Tukey's multiple comparisons test. NS: $P > 0.05$, *: $P \leq 0.05$, **: $P \leq 0.01$, ***: $P \leq 0.001$, ****: $P \leq 0.0001$.

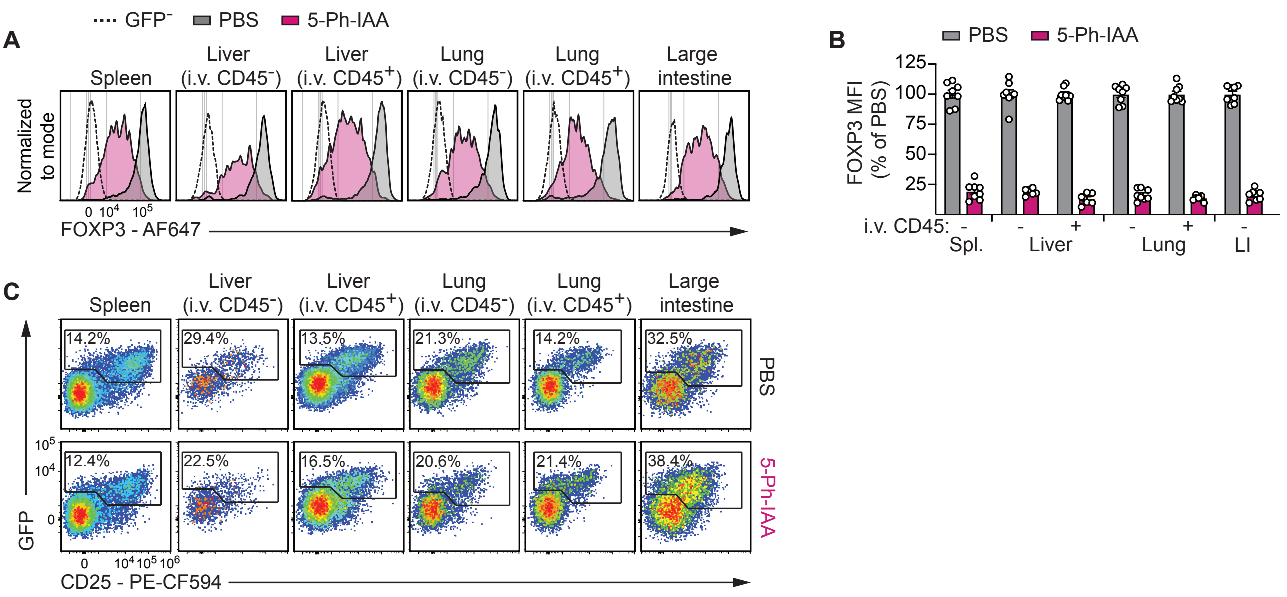

Fig. S2. FOXP3 protein degradation and CD25 levels across tissues.

(**A-B**) FOXP3 protein levels measured by intracellular staining against an endogenous FOXP3 epitope in GFP$^+$ CD4 T cells isolated from spleen, liver, lung, and large intestine lamina propria (LI) of 6-8 week-old male and female Foxp3AID mice injected daily with 5-Ph-IAA or PBS for 10 days. Cells were subdivided into those staining positive or negative for an intravenously injected fluorescent antibody against CD45 (i.v. CD45) to distinguish vascular-associated and extravascular immune cells, respectively. Panel B shows pooled data from 2 independent experiments with 6-8 biological replicates per condition. (**C**) Examples of GFP and CD25 staining on CD4 T cells isolated from the indicated organs.

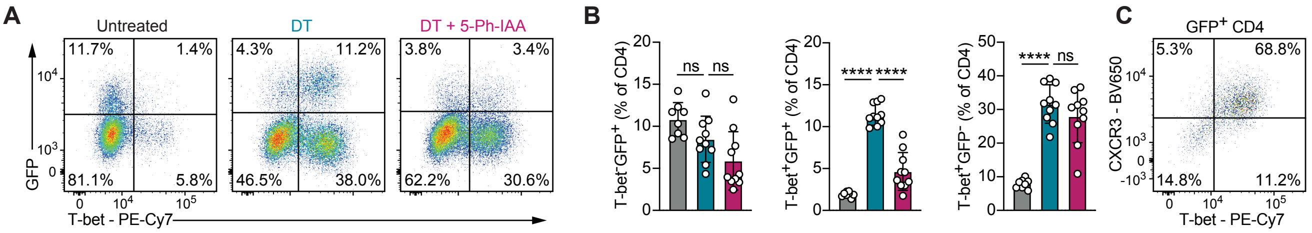

Fig. S3. FOXP3 protein degradation selectively depletes T-bet^{+} TREG cells.

(**A-B**) GFP and T-bet staining on splenic CD4 T cells on day 12 after transient TREG cell depletion. Related to Fig. 3I-J. Pooled data from two independent experiments with 8-10 mice per group. (**C**) T-bet and CXCR3 mark the same population of TREG cells. Representative staining from a DT-treated animal, gated on GFP^{+} CD4 T cells. Error bars show mean with SEM. *P* values were calculated using one-way ANOVA with Tukey's multiple comparisons test. NS: $P > 0.05$, *: $P \leq 0.05$, **: $P \leq 0.01$, ***: $P \leq 0.001$, ****: $P \leq 0.0001$.

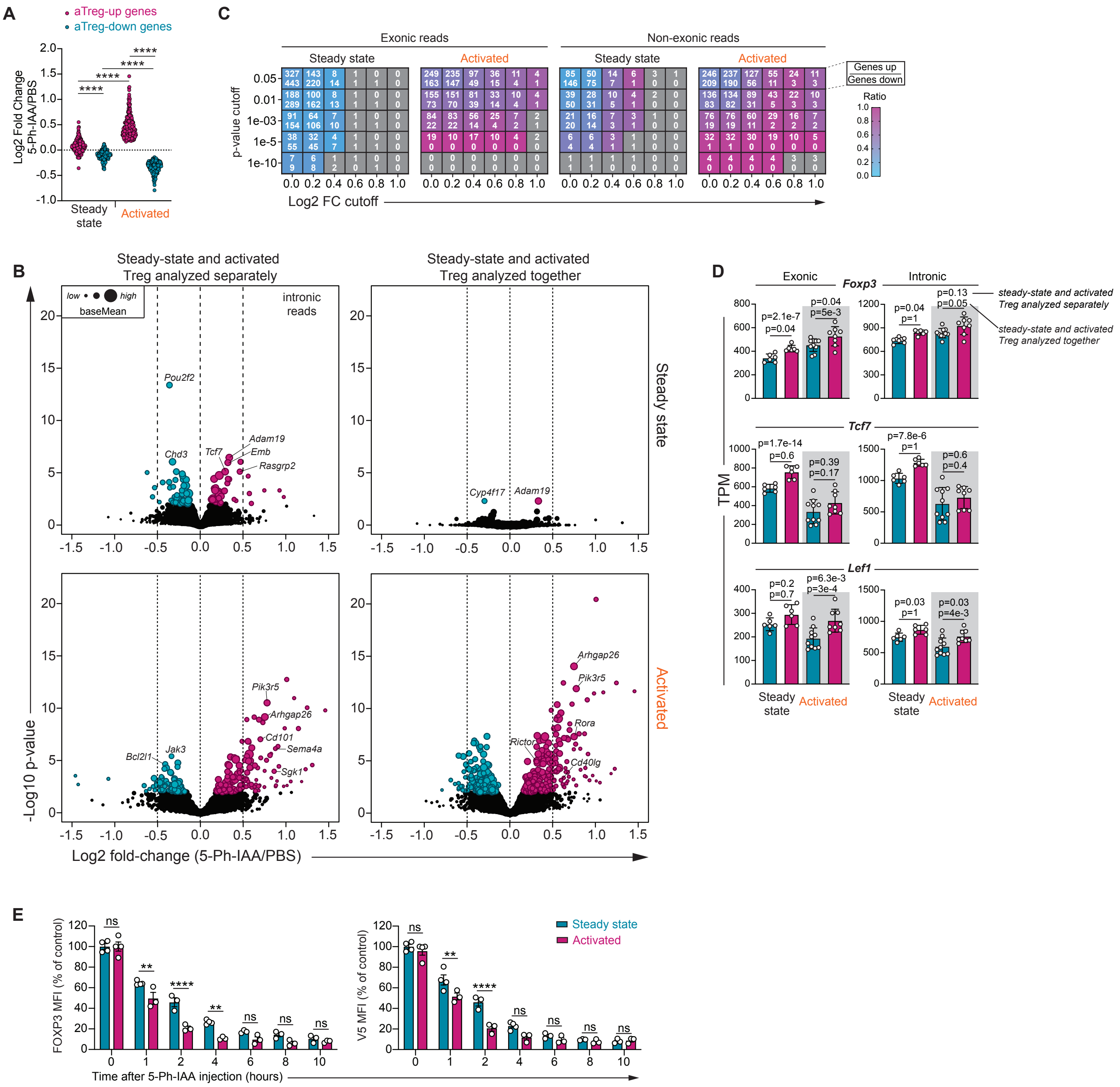

Fig. S4. RNA-seq analysis following FOXP3 protein degradation.

(**A**) Expression of the indicated differentially expressed gene sets in activated TREG (aTreg) and steady state TREG cells. Each dot indicates a gene. (**B**) Volcano plots showing differentially expressed genes (adjusted *P* value <0.01) when steady-state and activated TREG cells were analyzed separately (left) or together (right). (**C**) Differentially expressed genes at different p-value and log2 fold-change (FC) cutoffs, using exonic or intronic reads with steady-state and activated TREG cells analyzed separately. (**D**) Examples of differentially-expressed genes showing *P* values from the two different DESeq2 analyses. (**E**) FOXP3 protein degradation kinetics in steady-state and activated TREG cells. Pooled data from 2 independent experiments with a total of 3-4 mice per time point per condition. Error bars show mean with SEM. *P* values in panels A and E were calculated using one-way ANOVA with Tukey's multiple comparison's

test or two-way ANOVA with Šídák's multiple comparisons test, respectively. NS: $P > 0.05$, *: $P \leq 0.05$, **: $P \leq 0.01$, ***: $P \leq 0.001$, ****: $P \leq 0.0001$.

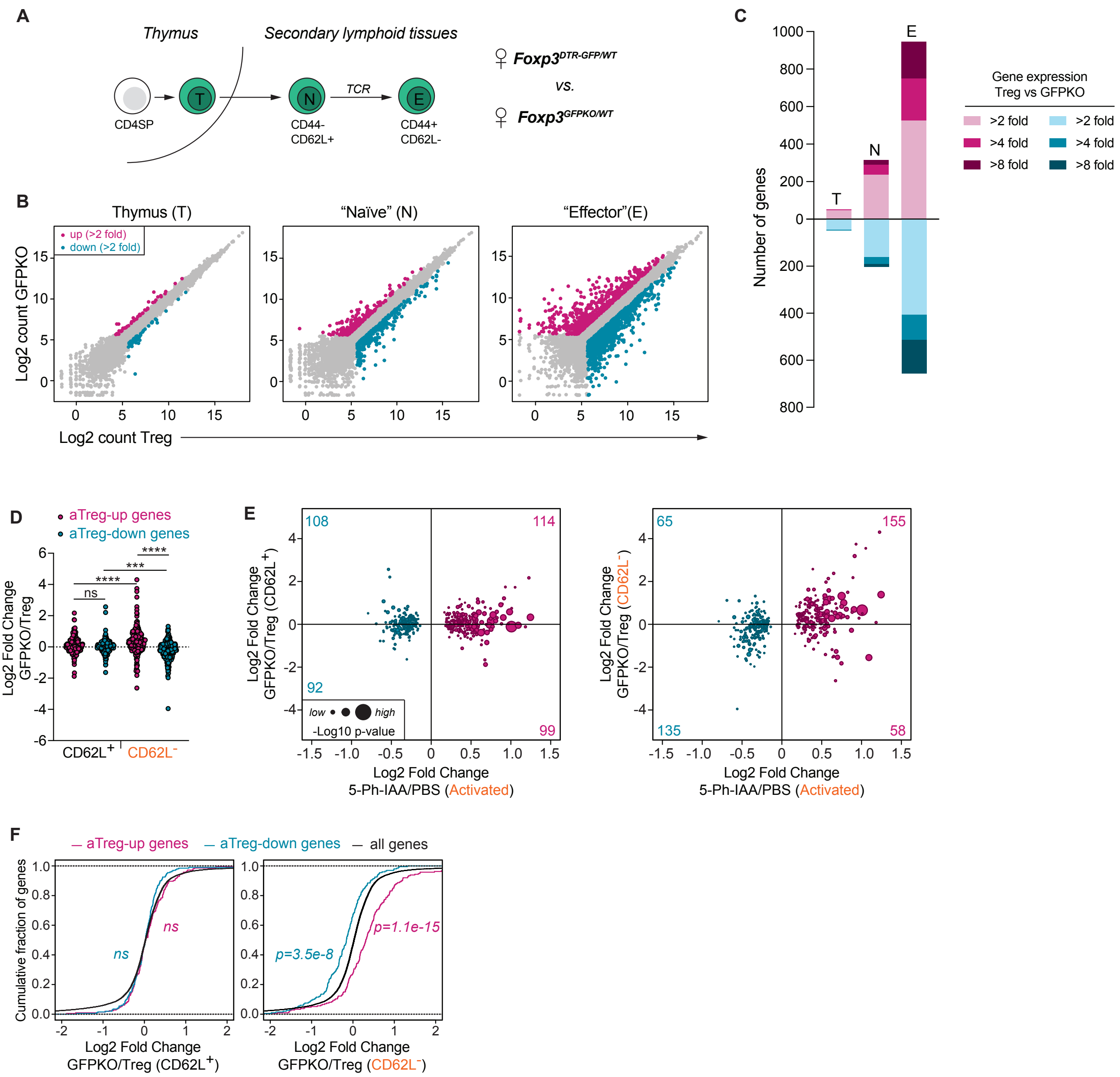

Fig. S5. Transcriptional changes induced by FOXP3 depletion or genetic *Foxp3* deficiency. (**A-B**) RNA-sequencing data from PMID33176163 and GSE154680. Treg and GFPKO cells were isolated from healthy heterozygous female *Foxp3*$^{DTR\text{-}GFP/WT}$ and *Foxp3*$^{GFPKO/WT}$ mice, respectively. Cells were compared at different stages of their development: as CD73^{-} CD4SP thymocytes (T), as CD62L^{+} "naïve" cells (N), or as CD62L^{-} "effector" cells (E). (**C**) Number of differentially expressed genes at different fold-change cutoffs is shown. (**D-F**) Expression of the indicated gene sets in CD62L^{+} or CD62L^{-} TREG versus GFPKO cells. *P* values from one-way ANOVA (D) or one-sided Kolmogorov-Smirnov test (F). NS: $P > 0.05$, *: $P \leq 0.05$, **: $P \leq 0.01$, ***: $P \leq 0.001$, ****: $P \leq 0.0001$.

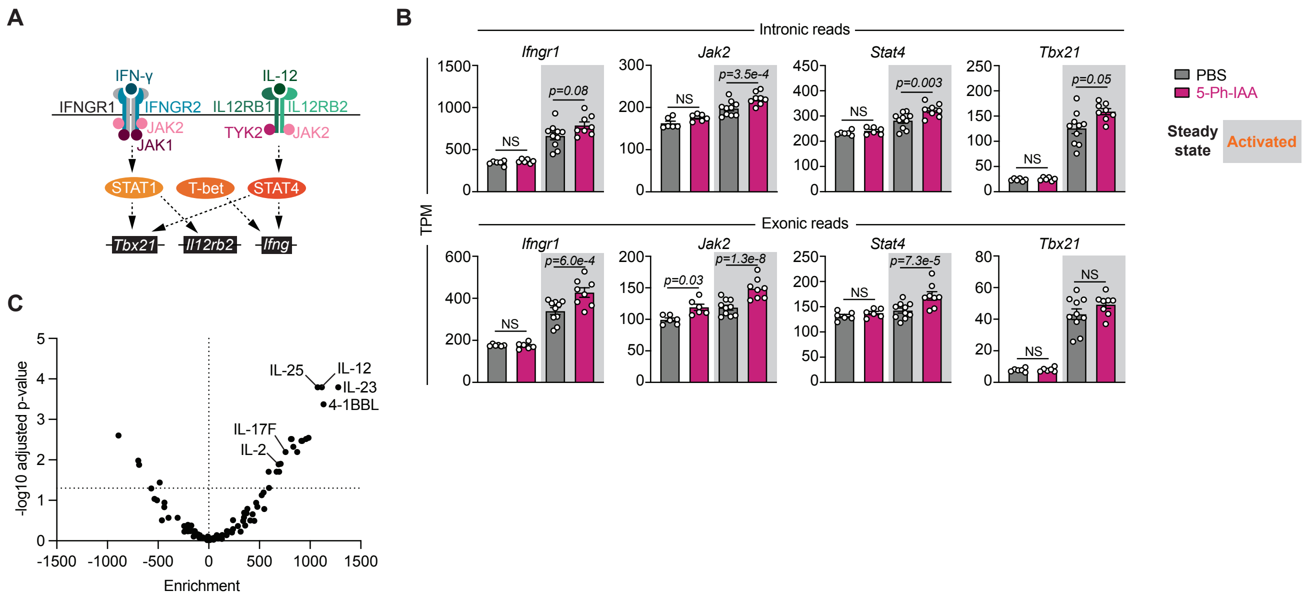

Fig. S6. FOXP3 directly represses genes involved in T_H1 differentiation.

(**A**) Schematic overview of signaling pathways activated during T_H1 cell differentiation. (**B**) Expression changes in *Ifngr1*, *Jak2*, *Stat4*, and *Tbx21* following acute FOXP3 protein degradation in steady-state versus activated T_{REG} cells (related to Fig. 4). (**C**) A previously published single-cell RNA-seq compendium of immune cell responses to 86 different cytokines (www.immune-dictionary.org) was used to perform Immune Response Enrichment Analysis (IREA) on the genes acutely upregulated following FOXP3 protein degradation in activated T_{REG} cells. Briefly, each dot represents a different cytokine that was injected subcutaneously into mice. Immune cell populations in the draining lymph node were then isolated and analyzed by single cell RNA-sequencing 4 hours later. The analysis shows how strongly the genes directly repressed by FOXP3 are induced in conventional CD4 T cells in response to each cytokine.

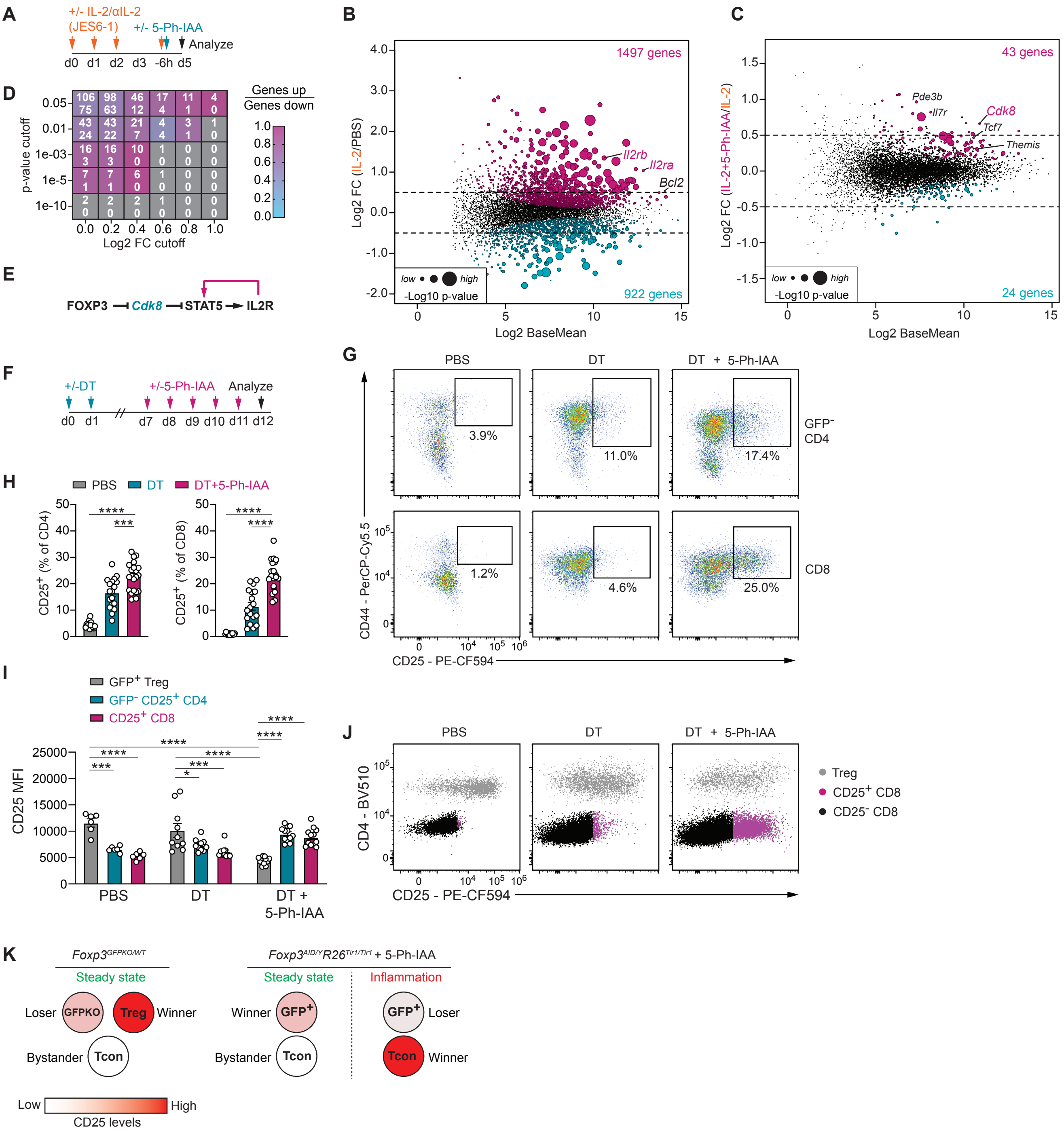

Fig. S7. FOXP3 regulates IL-2 responsiveness.

(**A**) Experimental design schematic. (**B**) Differentially expressed genes ($P < 0.01$) in GFP^{+} TREG cells isolated from secondary lymphoid tissues of mice injected with either PBS or IL-2/anti-IL2 (JES6-1) complexes. Data from intronic reads are shown. (**C-D**) Differentially expressed genes in GFP^{+} TREG cells isolated from mice injected with IL-2/anti-IL2 complexes with or without 5-Ph-IAA. (**E**) Proposed role of Cdk8 in regulation of the IL-2 signaling pathway. (**F**) Experimental design schematic. Related to Fig. 3E. (**G-I**) CD44 and CD25 levels on splenic GFP^{-} CD4 and CD8 T cells from the indicated treatment groups. Panel H shows pooled data from four experiments with 14-20 replicates per group. Panel I shows pooled data from two experiments with 6-12 replicates per group. (**J**) Overlay of GFP^{+} (grey) TREG cells,

$CD25^+$ CD8 T cells (purple) and $CD25^-$ CD8 T cells (black) from the indicated treatment groups. Panels G-J show pooled data from two independent experiments with 5-12 mice per group. (**K**) Model of T_{REG} cell competition for IL-2. In healthy heterozygous female *Foxp3*$^{GFPKO/WT}$ mice, wildtype T_{REG} cells outcompete reporter-null cells for IL-2. Under steady-state conditions, FOXP3-depleted GFP^+ T_{REG} cells express higher levels of CD25 than conventional CD4 and CD8 T (T_{CON}) cells and are therefore maintained. Under inflammatory conditions, activated T_{CON} cells upregulate CD25 and outcompete FOXP3-depleted Treg cells. Naïve T_{CON} cells in steady-state animals do not express CD25 or depend on IL-2, therefore acting as bystanders. Error bars show mean with SEM. *P* values were calculated using one-way (H) or two-way (I) ANOVA with Tukey's multiple comparisons test. NS: $P > 0.05$, *: $P \leq 0.05$, **: $P \leq 0.01$, ***: $P \leq 0.001$, ****: $P \leq 0.0001$.

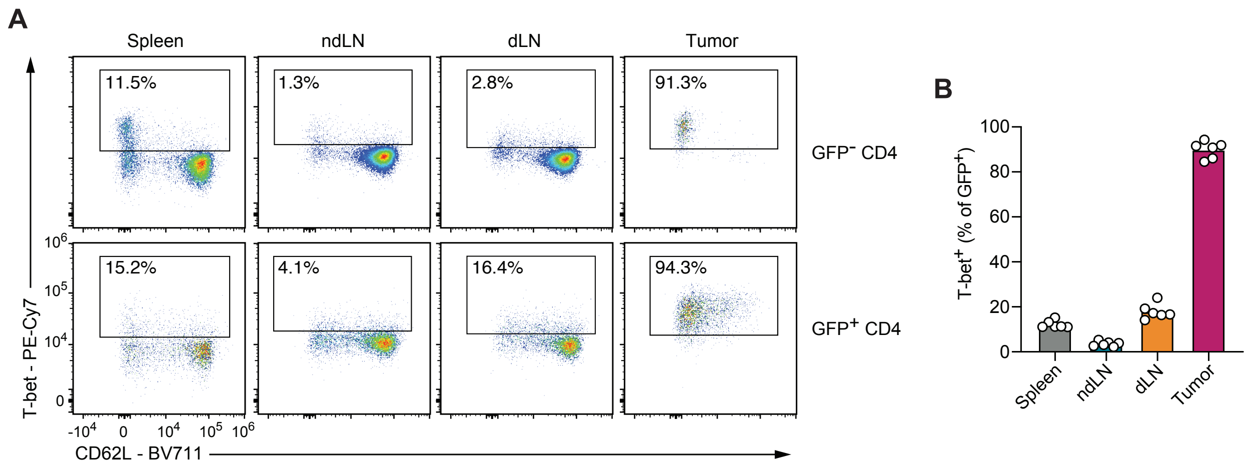

Fig. S8. Tumor-infiltrating T_{REG} cells express T-bet.

(**A-B**) T-bet levels in GFP^{+} and GFP^{-} CD4 T cell populations from spleens, non-draining lymph nodes, tumor-draining lymph nodes, and tumors of MC38 tumor-bearing mice. Representative data from one of two independent experiments with 6 mice. Error bars show mean with SEM.

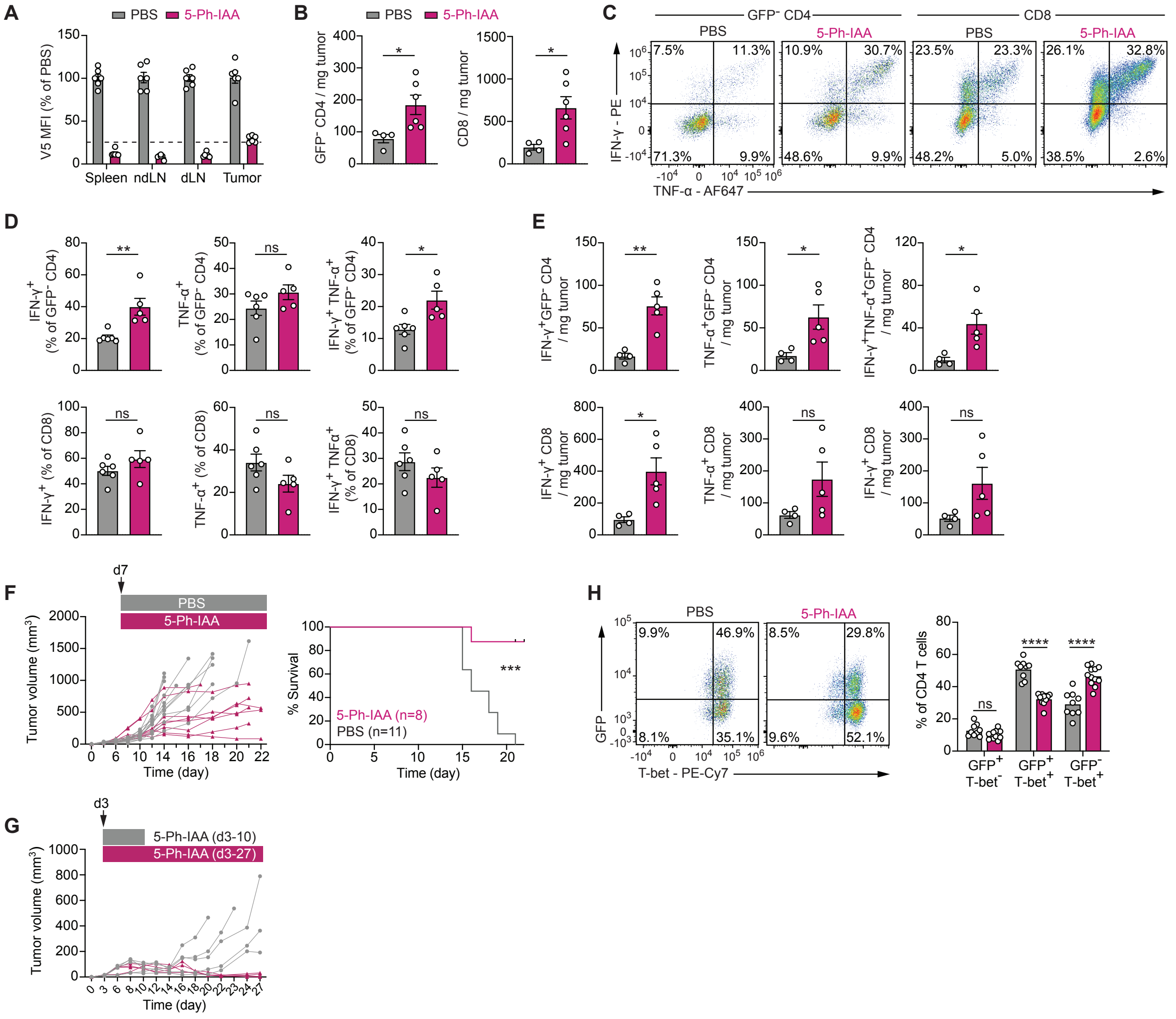

Fig. S9. FOXP3 degradation induces anti-tumor immune response.

(**A**) Tumor-bearing *Foxp3*^{AID} mice were injected with 5-Ph-IAA. 15 hours later, FOXP3 protein levels in GFP$^+$ TREG cells were quantified using intracellular antibody staining and flow cytometry. Pooled data from 2 independent experiments with a total of 6 mice per group. (**B**) GFP$^-$ CD4 and CD8 T cell counts in tumors isolated on day 11 after tumor implantation. Animals were treated daily with 5-Ph-IAA or PBS from day 3 onwards. (**C**) Example FACS plots of cytokine production by tumor-infiltrating T cells. (**D-E**) Frequency and total number of cytokine producing cells. Panels B-E show data from one of two independent experiments with 4-6 mice per group. (**F**) Tumor-bearing mice were treated with 5-Ph-IAA or vehicle control daily from day 7 onwards. Pooled data from two independent experiments with a total of 8 or 11 mice per group. (**G**) Tumor-bearing mice were treated daily with 5-Ph-IAA from days 3-10 or days 3-27. Pooled data from 2 independent experiments with a total of 4-5 mice per group. (**H**) T-bet levels in tumor-infiltrating CD4 T cells isolated from mice treated daily 5-Ph-IAA or PBS from day 3 onwards, as described in Fig. 6A. Pooled data from two independent experiments with a total of 9-12 mice per group. Error bars show mean with SEM. *P* values from unpaired *t* test (panels B, D, E), log-rank test (panel F), or two-way ANOVA

with Šidák's multiple comparison test (panel H). NS: $P > 0.05$, *: $P \leq 0.05$, **: $P \leq 0.01$, ***: $P \leq 0.001$, ****: $P \leq 0.0001$.
